# Problematic internet use as an age-related multifaceted problem: Evidence from a two-site survey

**DOI:** 10.1016/j.addbeh.2018.02.017

**Published:** 2018-06

**Authors:** Konstantinos Ioannidis, Matthias S. Treder, Samuel R. Chamberlain, Franz Kiraly, Sarah A. Redden, Dan J. Stein, Christine Lochner, Jon E. Grant

**Affiliations:** a Department of Psychiatry, University of Cambridge, UK; b Cambridge and Peterborough NHS Foundation Trust, Cambridge, UK; c School of Psychology, University of Birmingham, UK; d University College London, Department of Statistical Science, London, UK; e Department of Psychiatry and Behavioral Neuroscience, University of Chicago, Chicago, IL, USA; f SU/UCT MRC Unit on Risk and Resilience in Mental Disorders, Department of Psychiatry and Mental Health, University of Cape Town, South Africa; g SU/UCT MRC Unit on Risk and Resilience in Mental Disorders, Department of Psychiatry, University of Stellenbosch, South Africa

**Keywords:** Internet addiction, Behavioral addiction, Internet gaming disorder, Problematic internet use, Lasso, Machine learning

## Abstract

**Background and aims:**

Problematic internet use (PIU; otherwise known as Internet Addiction) is a growing problem in modern societies. There is scarce knowledge of the demographic variables and specific internet activities associated with PIU and a limited understanding of how PIU should be conceptualized. Our aim was to identify specific internet activities associated with PIU and explore the moderating role of age and gender in those associations.

**Methods:**

We recruited 1749 participants aged 18 and above via media advertisements in an Internet-based survey at two sites, one in the US, and one in South Africa; we utilized Lasso regression for the analysis.

**Results:**

Specific internet activities were associated with higher problematic internet use scores, including general surfing (lasso β: 2.1), internet gaming (β: 0.6), online shopping (β: 1.4), use of online auction websites (β: 0.027), social networking (β: 0.46) and use of online pornography (β: 1.0). Age moderated the relationship between PIU and role-playing-games (β: 0.33), online gambling (β: 0.15), use of auction websites (β: 0.35) and streaming media (β: 0.35), with older age associated with higher levels of PIU. There was inconclusive evidence for gender and gender × internet activities being associated with problematic internet use scores. Attention-deficit hyperactivity disorder (ADHD) and social anxiety disorder were associated with high PIU scores in young participants (age ≤ 25, β: 0.35 and 0.65 respectively), whereas generalized anxiety disorder (GAD) and obsessive-compulsive disorder (OCD) were associated with high PIU scores in the older participants (age > 55, β: 6.4 and 4.3 respectively).

**Conclusions:**

Many types of online behavior (e.g. shopping, pornography, general surfing) bear a stronger relationship with maladaptive use of the internet than gaming supporting the diagnostic classification of problematic internet use as a multifaceted disorder. Furthermore, internet activities and psychiatric diagnoses associated with problematic internet use vary with age, with public health implications.

## Highlights

• This is the first comprehensive exploration of various types of internet activities associated with maladaptive internet use.
• A range of activities beyond online gaming contributes separately and uniquely to problematic internet use.
• Specific internet activities are associated with more problems and that this relationship is influenced by age.

## 1 Introduction

Problematic internet use (PIU; otherwise known as Internet Addiction), is a public health concern in modern societies across the globe. The epidemiology of PIU is still unclear (Ho et al., 2014; Ko, Yen, Yen, Chen, & Chen, 2012) with a wide range of reported point prevalence estimates (1% to 36.7%), likely reflecting not only population differences but also the diversity of assessment tools and different operational definitions of PIU behaviors. DSM-5 has highlighted Internet gaming disorder as a condition for further study (American Psychiatric Association, 2013), specifically excluding other internet based activities like gambling and use of social media, despite the accumulating evidence that problematic internet use is a multifaceted problem that goes beyond online gaming (Király et al., 2014; Kuss & Lopez-Fernandez, 2016; Lopez-Fernandez, 2015). Many different online behaviors have been described as being capable of impairing normal functioning when undertaken to excess, including online gaming and massively-multiplayer online role-playing-games (Achab et al., 2011; Cole & Hooley, 2013; Ko, Yen, Yen, Lin, & Yang, 2007; Lecardeur, 2013; Lopez-Fernandez, 2015; Masten & Tellegen, 2012), online gambling (Griffiths, 2003; King & Barak, 1999), online shopping (Claes, Müller, & Luyckx, 2016; Rose & Dhandayudham, 2014; Trotzke, Starcke, Müller, & Brand, 2015), viewing pornography (Brand et al., 2011; King, 1999; Laier, Pawlikowski, Pekal, Schulte, & Brand, 2013), frequent email checking, instant messaging (Igarashi, Motoyoshi, Takai, & Yoshida, 2008; Rutland, Sheets, & Young, 2007; Yuen, Lavin, Weinman, & Kozak, 2004) and overuse of social media (Kuss & Griffiths, 2011; Kuss, Griffiths, & Binder, 2013). Online behaviors can also cause concern for individuals' physical health (Király, Griffiths, & Demetrovics, 2015; Tam & Walter, 2013) or lay the ground for criminal acts (Recupero, 2008). Impulsive and compulsive characteristics may underpin problematic internet behaviors (Block, 2008; Cao, Su, Liu, & Gao, 2007; Claes et al., 2016; Ioannidis et al., 2016; Trotzke et al., 2015), while specific internet activities have been linked with psychiatric disorders; for example, online shopping has been linked with depression and hoarding (Claes et al., 2016).

Young people and students are considered to be most vulnerable for PIU (Bakken, Wenzel, Götestam, Johansson, & Oren, 2009; Janower, 2006; Kuss et al., 2013; Kuss & Lopez-Fernandez, 2016; Wallace, 2014), but middle-aged and older populations have not been comprehensively investigated. Young age has been associated with problematic online shopping (Mueller et al., 2010; Trotzke et al., 2015). However, there have been a number of studies identifying problematic internet activities, including excessive internet based shopping, in adult populations (Rose & Dhandayudham, 2014). Overall, the natural history of problematic internet use is still unknown and there may be age related differences in PIU overall, or in different problematic online behaviors.

PIU has been considered to have a male preponderance (Shaw & Black, 2008; Tsai et al., 2009) and is likely more prevalent among Asian male youth, but females can also be vulnerable (Ha & Hwang, 2014; Xin et al., 2018). On a clinical level, the majority of PIU studies included male participants only (Kuss & Lopez-Fernandez, 2016) and it is unclear whether female clinical populations may have been understudied. There is some evidence from observational studies that males and females differ in the way they operate in the online environment in terms of activities they choose and their negative consequences (Ha & Hwang, 2014; Liang, Zhou, Yuan, Shao, & Bian, 2016). Excessive use of chatting and social media has been associated with female gender in young students (Andreassen, Torsheim, Brunborg, & Pallesen, 2012; Fernández-Villa et al., 2015; Kittinger, Correia, & Irons, 2012; Kuss & Griffiths, 2011; S Laconi, Andréoletti, Chauchard, Rodgers, & Chabrol, 2016). Female gender has also been identified as a predictor of problematic online shopping (Rose & Dhandayudham, 2014), but the opposite has also been reported (Fernández-Villa et al., 2015; Laconi et al., 2006). Online gaming has been associated with male gender (Fernández-Villa et al., 2015), but massively multiplayer online role-play gaming has been reported in both genders (Achab et al., 2011). Online pornography as well as online gambling have been reported to be more frequent among adult males (Laconi et al., 2016), however, it has been argued that the role of reward reinforcement, cue reactivity and craving of online sex are similar for both genders (Laier et al., 2013). Particular platforms of social media with addictive potential, such as networking sites like Facebook, are used by both genders and it has been argued that females might be particularly at risk (Kuss et al., 2013). Overall, there might be gender-specific differences for aspects of PIU; alternatively, it may be that once clinical and behavioral characteristics/confounds are taken into account, both genders are similarly affected (Ioannidis et al., 2016; Király et al., 2014; Kuss et al., 2013).

Overall, problematic internet use including the wide variety of problematic internet behaviors require more rigorous investigations that would shed light onto which specific activities should be regarded as problematic or dysfunctional or in general contributing to the phenomenon described as PIU. The way in which age and gender moderate the relationship between particular internet activities and PIU has been understudied, warranting more attention.

Our objective was to identify specific internet-related activities statistically associated with PIU and whether there are interactions with age or gender that moderate those relationships.

## 2 Material and methods

### 2.1 Setting and measures

More details about the setting and measures of this study have also been described in our previous publication on PIU (Ioannidis et al., 2016). Reporting of methods for this study follows the STROBE guideline (von Elm et al., 2008). The current study was conducted from January 2014–February 2015. Individuals aged 18 years and above were recruited at two sites: Chicago (USA) and Stellenbosch (South Africa) using internet advertisements (mean age 29 [18–77]; 1119 males [64%]; 1285 Caucasian [73%]). The advertisements asked individuals to take part in an online survey about internet use. Participants completed the survey anonymously using Survey Monkey software. The survey was sent through Craigslist so only participants from the specific locales were targeted. The study was approved by the institutional review boards at each research site. Participants received no compensation for taking part but were enrolled in a random lottery whereby five prizes were available with each prize valued between $50 and $200 in USA and three prizes between ZAR250 and ZAR750 in South Africa.

The online survey contained questions about each individual's age, gender, race, relationship status, sexual orientation and education background, along with various measures of specific internet activities. We measured a number of different internet activities including 1) general surfing 2) internet gaming total 3) Online role playing games (RPG) 4) Time wasters/skill games (i.e. Apps on iPod/iPad/cell phone, Tetris, Jewels) 5) Online action multiplayer (i.e. Call of Duty, Gears of War) 6) Online shopping 7) Auction websites (i.e. Ebay) 8) Online gambling 9) Social networking 10) Online sports (i.e. Fantasy sports, ESPN) 11) Pornography/Sex on internet 12) Messaging/Blogging (i.e. AIM, Skype) and 13) Streaming videos/media (i.e. YouTube, Hulu). The survey also included clinical measures: the Internet Addiction Test (IAT) (Young, 1998) to provide a measure of maladaptive internet use; select Mini International Neuropsychiatric Interview (MINI) modules (Sheehan et al., 1998) to identify probable social anxiety disorder (SAD), generalized anxiety disorder (GAD) and obsessive-compulsive disorder (OCD); the Adult ADHD Self-Report Scale Symptom Checklist (ASRS-v1.1) (Kessler et al., 2005) to identify attention-deficit hyperactivity disorder (ADHD) symptoms; the Padua Inventory (PI) (Burns, Keortge, Formea, & Sternberger, 1996) to identify obsessive-compulsive tendencies; and the Barratt Impulsiveness Scale (BIS-11) to quantify impulsive personality (Patton, Stanford, & Barratt, 1995). Descriptive statistics for all variables are summarized and stratified by age in Supplementary Table S1a.

The IAT comprises 20 questions examining facets of PIU. Scores on the IAT range from 20 to 100 with 20–49 reflecting mild Internet use, 50–79 moderate Internet use, and 80–100 reflecting severe Internet use. The PI consists of 39 items assessing common obsessional and compulsive behavior. The BIS-11 is a self-report questionnaire used to determine levels of impulsiveness.

We performed a Principal Components Analysis (PCA) to identify whether a few components of internet activities would be able to account for a significant portion of the variance. However, this analysis showed that we required >11 out of 13 components to achieve >90% of variance indicating that a significant portion of the variables of internet activities contribute uniquely to the variance. We therefore decided to use each variable separately in our analysis.

**Fig. 1:**
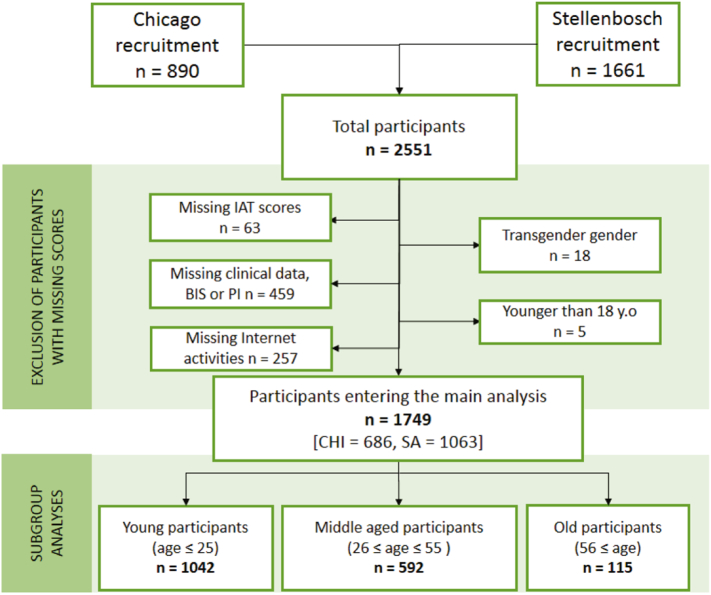
Recruitment flow diagram. Flow diagram describing recruitment and exclusion from main and subgroup analyses; IAT: Internet Addiction test; PI: Padua Inventory-Revised; BIS - Barratt Impulsiveness Scale 11; CHI – Chicago; SA – South Africa (Stellenbosch). (For interpretation of the references to colour in this figure legend, the reader is referred to the web version of this article.)

Only data from participants who completed the entirety of the online survey, including the internet activity measures, were included in the analyses. The original sample included 2551 individuals. 63 individuals were excluded for lacking IAT scores. A further 18 individuals were excluded for reporting transgender gender and 459 for missing important predictor variables e.g. PI or BIS questionnaire scores. Five individuals were excluded for reporting age <18 years old. A further 257 individuals were excluded due to missing measures of internet activity. The final full set included 1749 individuals with complete scores on all variables. This last step of exclusion process accounts for the sample difference between the present study and Ioannidis et al., 2016. This final full set included 1063 individuals from the Stellenbosch site and 686 individuals from the Chicago site. The estimated point prevalence of PIU was ~8.5% using an IAT cut-off of 50 or above. Comparing the two study site populations, the Stellenbosch site had younger participants [mean(range) 24.3(18–76) vs 36.3(18–77), ANOVA F < 0.05, *η*^2^: 0.20], a lower proportion of male gender [58% vs 73%, *χ*^2^ < 0.05, *φ*:0.15], higher proportion of heterosexual sexual orientation [91% vs 84%, *χ*^*2*^ < 0.05, *φ*:0.10], higher rates of ADHD [50% vs 41% *χ*^*2*^ < 0.05, *φ*:0.9], lower rates of online shopping [mean(range) 0.48(0–5) vs 1.27(0–5), ANOVA F < 0.05, *η*^2^: 0.18] and slightly lower IAT scores [mean(range) 30.3(20–94) vs 35.9(20–85), ANOVA F < 0.05, *η*^2^: 0.06]. A more detailed comparison is presented in the Supplementary Table S1b. Recruitment and exclusion process are graphically presented in Fig. 1. All continuous variables (i.e. BIS score) were standardized to increase the interpretability of the model coefficients. The prediction methods used the IAT score as a numeric variable (Range 20–94, Mean 32.48). All analyses were undertaken in R Studio version 3.1.2. Lasso Generalized Linear Models were performed using the “glmnet” package (Package glmnet version 2.0–5 (Friedman, Hastie, & Tibshirani, 2010)). More details about the analysis process can be found in the Supplement (methodology appendix).

### 2.2 Exploration of correlations

**Fig. 2:**
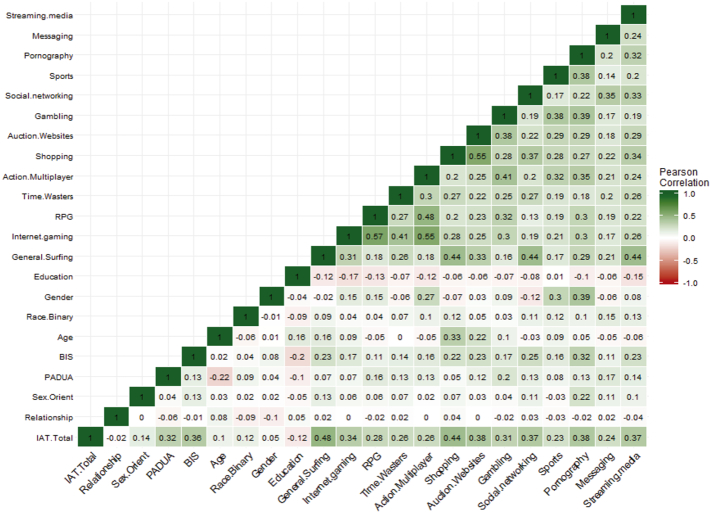
Exploratory correlation matrix of variables. Pearson correlations between all variables. Positive correlations are indicated in green gradient colour, negative correlations are in red gradient. IAT. Total - Internet Addiction Score; PADUA - PADUA Inventory score; BIS - Barratt Impulsiveness Scale score; RPG - Online Role Playing games. (For interpretation of the references to colour in this figure legend, the reader is referred to the web version of this article.)

We explored correlations between the variables in our data (see Fig. 2). All different internet activities had weak positive correlations with the IAT score (Pearson correlation coefficient range 0.23–0.48). Some moderate positive correlations between internet activity variables were identified i.e. total internet gaming and RPG (r = 0.57), total internet gaming and action multiplayer games (r = 0.55), online shopping and use of auction websites (r = 0.55), general surfing and shopping (r = 0.44), general surfing and social networking (r = 0.44), general surfing and streaming media (r = 0.44). There were weak positive correlations between sports and pornography (r = 0.38), male gender and sports (r = 0.30) or pornography (r = 0.39) or action multiplayer gaming (r = 0.27). There were weak correlations between online gambling and action multiplayer (r = 0.41), RGP (r = 0.32), auction websites (r = 0.38), sports (r = 0.38) or pornography (r = 0.39). Impulsivity was weakly positively correlated with general surfing, online shopping, use of auction websites, social networking, streaming media and pornography (0.2 ≤ r ≤ 0.3). There was also a weak correlation between older age and shopping activities (r = 0.33) or use of auction websites (r = 0.22), and between non-heterosexual sexual orientation and pornography (r = 0.22). All other correlations between internet activities and age, gender, relationship status, sexual orientation, level of education, race and levels of impulsivity and compulsivity were very weak (−0.2 < r < 0.2).

### 2.3 Dealing with over-fitting

**Fig. 3:**
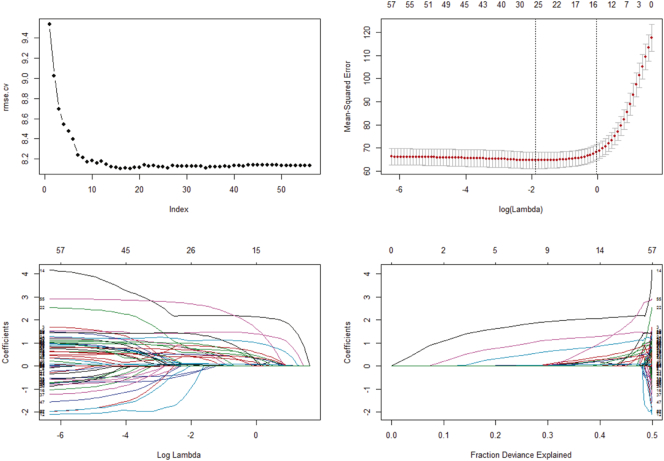
Explanatory plots for cross-validated errors and Lasso coefficients. Explanatory plots for cross-validated errors and Lasso coefficients (all participants n = 1749). The first plot (top left) demonstrates the cross-validated root mean squared error (rmse.cv) as a function of number of variables included in the linear regression model. The plot demonstrates that adding more than ~16 variables in the model does not necessarily improve the model in terms of RMSE reduction. The second plot (top right) demonstrates the 10-fold cross-validated mean squared error as a function of (log) lambda (λ) for the lasso regularized model using the full data with interaction terms. The top numbering of the plot indicates the number of predictors (variables) the model is using, going from all predictors (top left corner) to more sparse models (top right corner). This function helps the optimization of Lasso in terms of choosing the best λ. The third plot (bottom left) shows the predictors coefficients scores as a function of log(λ) indicating the shrinkage of coefficients for larger numbers of log(λ). The top numbering of the plot indicates the number of predictors (variables) the model is using, going from all predictors (top left corner) to more sparse models (top right corner). The last plot (bottom right) shows the fraction of deviance explained by the models in relation to the number of predictors used and their coefficients. Each coloured line described a single predictor and its coefficient score. The plot shows that close to the maximum fraction of deviance explained larger coefficients occur indicating likely over-fitting of the model. (For interpretation of the references to colour in this figure legend, the reader is referred to the web version of this article.)

For our statistical methods we used models that included demographic variables (age, race, education level, gender, relationship status, sexual orientation), clinical characteristics (diagnoses of ADHD, GAD, Social Anxiety and OCD), behavioral dimensions known to be associated with PIU (impulsivity and compulsivity), internet activities and interaction terms between Internet activities × Age or Gender; the latter was decided to test the hypothesis that age or gender moderate the relationship between internet activities and problematic internet use scores. We included a total of 51 predictor variables. By including a plethora of variables we aimed for a model that is more accurate and at the same time captures complex interactions between demographic and internet activity variables. However, the downside of having many predictor variables is that this typically leads to over-fitting accompanied by large coefficients. Furthermore, in-sample linear regression also tends to over-fit, especially in complex models, and is fundamentally flawed in making predictions on new data. There is extensive evidence of the downsides of over-fitting models (Huys, Maia, & Frank, 2016). To deal with over-fitting, we have discussed using out-of-sample statistical methods (cross-validation) to get an estimate of the generalization and prediction error of the model (Ioannidis et al., 2016). We explored this approach in our current data when we used an out-of-sample cross-validated estimation of the root-mean-squared-error in conjunction with backward selection of variables to test whether models improve by adding a high number of variables in the subsets of possible combinations of predictors, and we saw that sparse models (i.e. with about between 13 and 16 variables) were non-inferior in terms of cross-validated RMSE compared to more complex models (including >16 variables). This is shown in exploratory Fig. 3 (top left).

### 2.4 Regularized regression with sparsity constraints

For the reasons mentioned in the previous paragraph, we wanted to use a method of prediction that would not over-fit as much, while being comparable to standard statistical methods in terms of predicting PIU scores. It would also be valuable if our method could also do variable selection (i.e. by reducing the number of predictors with non-zero coefficients), in order to help with the interpretability of the model. Regularization, initially designed by Tikhonov to solve integral equations (Tikhonov, 1963) and later introduced in statistical science by Tibshirani (1996) has some of the desired aforementioned properties of shifting model construction towards sparsity and reducing over-fitting (Huys et al., 2016). Lasso (generalized linear model with penalized maximum likelihood, known as regression using Least Absolute Shrinkage and Selection Operator (Lasso or LASSO (Tibshirani, 1996))) is a regularization and regression analysis method now often used in medical sciences (Bujak, Daghir-Wojtkowiak, Kaliszan, & Markuszewski, 2016; Kim, Kang, Biswas, Liu, & Gao, 2016) and has potential for use in clinical prediction modeling in psychiatry (R C Kessler et al., 2016). Ridge regression is another form of regularized linear regression that shrinks coefficients by introducing a coefficient penalty (Hoerl & Kennard, 1970). The elastic-net is an intermediate model between ridge and lasso and its penalty is controlled by α, which bridges the gap between Lasso (α = 1) and ridge (α = 0). The tuning parameter λ controls the overall strength of the penalty. Lasso uses the L1 penalty and ridge uses the L2 penalty. In contrast to ridge regression, the effect of the Lasso L1 penalty is that most coefficients are driven to zero, leading to a regularized solution that is sparse at the same time. By this mechanism, the Lasso performs variable selection which can greatly simplify interpretation especially if many predictors are involved in the model. Another non-standard method known for high accuracy and ability to avoid over fitting is random forests (Breiman, 2001). Random forests are a machine learning method that performs well against non-linear dependencies and therefore, exploring the performance of this model could give us insight into, possibly ‘hidden’, complex associations.

### 2.5 Prediction methods

To choose the appropriate model in our analysis, we compared linear regression, ridge regression, elastic-net, Lasso and random forest models with one another and against a naïve baseline, using a cross-validated out-of-sample estimate of RMSE. Our cross-validation included randomly splitting the data in a training and testing set, tuning the model parameters in the training set and making predictions for IAT scores in the testing set. Due to the random nature of splitting the data into folds, we repeated this process 50 times to get a stable and replicable estimate. We then compared the final vectors of RMSE scores using Exact Wilcoxon-Pratt signed rank tests. All models were significantly superior to the naïve baseline (p corrected <0.001, Cohen's d = −0.87) (see Supplementary Table S2). Summary statistics of RMSE scores are presented in Supplementary Table S3. Lasso and elastic net were superior to ridge regression (p-corrected <0.01, d = 0.51, d = 0.49) and linear regression (p corrected <0.001, d = 0.76) and not statistically different between each other (p corrected >0.05, d = −0.08). Random forest was non-superior to either lasso (p = 0.12) or elastic net (p corrected >0.05). Therefore, in our analysis, we used Lasso, because, further to good out-of-sample prediction performance, Lasso was able to perform variable selection by shrinking coefficients to zero and therefore increasing interpretability. Although the elastic net can also perform variable selection, it tends to select more variables, and despite being a more complex and more powerful model, it did not give significantly better performance than lasso. In our final analysis full data and subgroup analyses, we used 10-fold cross-validation to produce the optimal lambda for each lasso model and report coefficients produced by those models. Explanatory plots deriving from the full data analysis are presented in Fig. 3.

## 3 Results

Lasso regression results are summarized in the whole sample and stratified by age in Table 1, Table 2. Full tables of results for subgroup analyses, including stratified by age and by study site are presented in the online Supplementary tables (Tables S4–S10). Exploratory plots of the data are presented in Supplementary figures (Figs. S1–S3). Results from the more standard statistical approach of linear regression are also presented in Supplementary Tables S4–S10 and any differences in structural inference compared to the main results presented below are conditional upon the choice of another model.

**Table 1:** Lasso coefficients for internet activities stratified by age.

Internet activity	All (n = 1749)	18 ≤ Age ≤ 25 (n = 1042)	26 ≤ Age ≤ 55 (n = 592)	Age > 55 (n = 115)
General surfing	**2.100**	**2.400**	**1.500**	**0.590**
Internet gaming	**0.600**	**0.450**	**0.110**	0.000
RPG	0.000	0.000	**0.710**	0.000
Time wasters	0.000	0.000	0.000	**0.450**
Action multiplayer	0.000	0.000	0.000	0.000
Shopping	**1.400**	**0.840**	**1.500**	0.000
Auction websites	**0.027**	0.000	**0.990**	**0.230**
Gambling	0.000	0.000	**0.780**	0.000
Social networking	**0.460**	0.000	**1.300**	0.000
Sports	0.000	0.000	0.000	0.000
Pornography	**1.000**	**1.400**	**0.210**	0.000
Messaging	0.000	0.000	**0.110**	0.000
Streaming media	0.000	0.000	0.000	**1.200**
PADUA	**0.074**	**0.085**	**0.029**	**0.065**
BIS	**0.066**	**0.048**	**0.072**	**0.086**
ADHD Diagnosis	**1.700**	**0.350**	**3.100**	0.000
GAD diagnosis	**0.230**	0.000	0.000	**6.400**
Social anxiety diagnosis	0.000	**0.560**	0.000	0.000
OCD diagnosis	**0.270**	0.000	0.000	**4.300**

Lasso - least absolute shrinkage and selection operator; RPG - Role Playing games; PADUA: Padua Inventory-Revised Checking; BIS - Barratt Impulsiveness Scale 11; ADHD - Attention Deficit Hyperactivity Disorder; GAD – Generalized Anxiety disorder; OCD – Obsessive-Compulsive disorder. For presentation purposes the significant Lasso coefficients are indicated in bold.

**Table 2:** Lasso coefficients for demographics and interaction terms.

Internet activity	All (n = 1749)	18 ≤ Age ≤ 25 (n = 1042)	26 ≤ Age ≤ 55 (n = 592)	Age > 55 (n = 115)
Demographic variables	0.000	0.000	0.000	0.000
Gender × any Internet activity	0.000	0.000	0.000	0.000
Age × general surfing	0.000	–	–	–
Age × Internet gaming	0.000	–	–	–
Age × RPG	**0.330**	–	–	–
Age × time wasters	0.000	–	–	–
Age × action multiplayer	0.000	–	–	–
Age × shopping	0.000	–	–	–
Age × gambling	**0.150**	–	–	–
Age × auction websites	**0.350**	–	–	–
Age × social networking	0.000	–	–	–
Age × sports	0.000	–	–	–
Age × pornography	0.000	–	–	–
Age × messaging	0.000	–	–	–
Age × streaming media	**0.350**	–	–	–

Lasso - least absolute shrinkage and selection operator; RPG - Role Playing games; Demographic variables are: Age, Gender, Race, Education, Relationship status and Sexual Orientation. For presentation purposes the significant Lasso coefficients are indicated in bold.

### 3.1 Demographics

In lasso regression no variable including age, gender, race, education level, relationship status or sexual orientation was associated with PIU in any age subgroup or in the full data.

### 3.2 Internet activities

In the full data Lasso regression, a number of internet activities were associated with high PIU scores including general surfing (β: 2.1), internet gaming (β: 0.6), online shopping (β: 1.4), use of auction websites (β: 0.027), social networking (β: 0.46) and use of online pornography (β: 1.0). The relationships between PIU and role-playing-games (RPGs), online gambling, use of auction websites and using streaming media were moderated by age (β: 0.33, 0.15, 0.35 and 0.35 respectively), with older age associated with higher PIU scores. In age-subgroup analysis (young participants age ≤ 25, middle age participants 25 < age ≤ 55; older participants age > 55), general surfing was associated with PIU in all age groups, but more strongly in the young (β: 2.4), less in the middle aged (β: 1.5), and even less in the older participants (β: 0.59). A similar trend was seen in internet gaming (β: 0.45, 0.11 and 0.0 for the three age groups respectively) and use of online pornography (β: 1.4, 0.21 and 0.0). Some internet activities such as use of online RPGs were more strongly associated with PIU in middle aged participants compared to other age groups (β: 0.71). The same was true for online gambling (β: 0.78), instant messaging (β: 0.11) and online social networking (β: 1.3). Use of auction websites was also more strongly associated with PIU in middle aged participants (β: 0.99), but also predictive in the older participants (β: 0.23). Streaming online media and use of time wasters were associated with PIU in the older participants (β: 1.2, 0.45 respectively), but not in any other age group.

### 3.3 Clinical and behavioral characteristics

Symptoms of attention-deficit hyperactivity disorder (ADHD) (β: 1.7), generalized anxiety disorder (GAD) (β: 0.23) and obsessive-compulsive disorder (OCD) (β: 0.27) were associated with higher PIU scores. In age-subgroup analysis, ADHD and SAD were associated with higher PIU scores in younger participants (β: 0.35 and 0.56 respectively), while ADHD remained significant in the middle-aged subgroup (β: 3.1). GAD and OCD were associated with higher PIU scores in the older participants subgroup (β: 6.4 and 4.3 respectively), but not in the other age groups. BIS scores (impulsive personality) and PADUA scores (obsessive-compulsive tendencies) were associated with higher PIU scores in the full data (β: 0.066 and 0.074 respectively) and in all age subgroups analyses.

## 4 Discussion

This paper is the first attempt to comprehensively explore the different types of internet activities associated with maladaptive use of the internet, i.e. with problematic internet use. Previous work has generally tackled the issue of specific internet activities leading to problematic use by focusing on isolated internet activities (Achab et al., 2011; Claes et al., 2016; Griffiths, 2003; Ko et al., 2007; Kuss & Griffiths, 2011). We have shown here that a range of internet activities, including general surfing, internet gaming, online shopping, use of auction websites, online gambling, social networking and use of online pornography contribute separately and uniquely to PIU, providing evidence that PIU is a complex phenomenon comprising a variety of problematic behaviors. Further to that, we have shown that those behaviors retain their statistically significant associations with PIU, even when psychiatric symptoms known to be associated with PIU (i.e. symptoms of ADHD, GAD and OCD) (Carli et al., 2013; Ho et al., 2014) and dimensions of behavior known to be predictive of PIU (i.e. personality measures of impulsivity and compulsivity) (Cao et al., 2007; Claes et al., 2016; Ioannidis et al., 2016; Trotzke et al., 2015) are taken into account. We have further demonstrated that specific internet activities like RPG, online gambling, use of auction websites and streaming media are associated with higher PIU scores and that this relationship is influenced by age. Finally, our data show that other types of online behavior (e.g. shopping, pornography, general surfing) bear a stronger relationship with maladaptive use of the internet than gaming and it is possible that this relates to the fact that previous studies have not included such a wide array of internet-related activities. These results have significant implications for the conceptualization of PIU as a clinically meaningful disorder, as they draw the attention away from the unidimensional and relatively narrow construct of ‘Internet gaming disorder’, towards a multidimensional entity of problematic internet use or internet addiction comprising multiple facets of human online behavior.

Moreover, using out-of-sample cross validation we have shown that the ‘non-standard’ approach of using Lasso regression is more accurate in predicting PIU scores as compared to the ‘more standard’ linear regression. Using out-of-sample estimation of the predictive value of a model often helps with tackling the phenomenon by which significances decay in replication studies. However, the choice of Lasso regression comes with the caveat that variables which are not selected by the model (with zero coefficients) can still be predictive, especially when there are high correlations between selected and non-selected variables. In our data set, we did not have any highly correlated variables, nevertheless, this limitation means that we should treat any negative results conservatively. For example, the lack of association between gender and PIU as well as the lack of association between gender × Internet activities with PIU arguably supports the hypothesis that if a wider range of PIU behaviors and potential confounders are taken into account, both genders are equally vulnerable to developing facets of PIU (Ioannidis et al., 2016; Király et al., 2014). However, due to the limitations of our analysis, we cannot exclude the possibility that other associations between PIU and gender exist. For example, it has been suggested that gender moderates the relationship between online shopping and PIU and that females may be more at risk (Rose & Dhandayudham, 2014). Of relevance may be that compulsive buying disorder, a disorder that is prominent in middle aged groups has a female predominance by 5:1 ratio (Black, 2007), and may be driving such findings. We did not have any data on this disorder to test this hypothesis. It is also important to note, that the IAT instrument used here has received critique on its lack of robustness concerning factor structure, differences from current DSM-5 operationalization (gaming disorder) and lagging behind the technological advances of internet applications (Khazaal et al., 2015; Laconi, Rodgers, & Chabrol, 2014). Future PIU research would be well served by methodologically robust, validated instruments, which would also be able to capture the quickly evolving nature of PIU from a technological and behavioral perspective.

Our age-subgroup analysis provided insight into the age related associations between PIU and various internet activities. The common conception that PIU is a disorder of youth is not necessarily correct and may be based on the lack of appropriately designed studies that capture online behaviors across all age groups. Insufficient knowledge for the natural history of PIU across the lifespan does not allow a comprehensive exploration of vulnerabilities in the older populations in terms of risk to develop PIU. However, our results indicate that those vulnerabilities do exist and further research is warranted to map out the characteristics of the populations at risk. For example, having ADHD or social anxiety symptoms may be a predictor for PIU in young populations, whereas having OCD or GAD symptoms may be a predictor for PIU in older populations. The fact that OCD was not found to be associated with PIU in a recent meta-analysis (Ho et al., 2014) may be an indicator that older populations have been understudied. The fact that ADHD was strongly associated with high PIU scores is not surprising, as other studies have reported a very high prevalence of ADHD (up to 100%) in PIU populations (Carli et al., 2013). At the same time, specific middle aged populations (between 26 and 55) may be more at risk of PIU, if they also suffer from compulsive buying disorder or gambling disorder, given the natural history of those disorders, which peak in middle age (Cunningham-Williams et al., 2005).

**Fig. 4:**
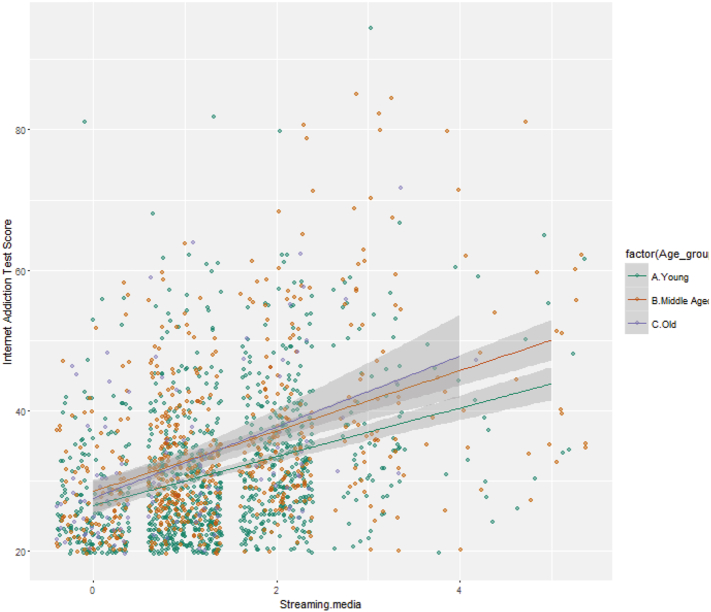
Example exploratory figure of the association between Problematic internet use and streaming media, by age group. This is an example figure showing the relationship between Problematic internet use (PIU) and streaming media grouped by age. The regression lines are linear models with confidence intervals (grey areas). Interestingly, streaming media appears to be less associated with PIU in the young age ≤ 25 as compared to older people >55 (also shown in Lasso analysis in the main paper; Lasso coef Streaming media β: 0.0 for young and β: 1.2 for old, Age × Streaming Media interaction Lasso coef β: 0.35). (For interpretation of the references to colour in this figure legend, the reader is referred to the web version of this article.)

Furthermore, the findings that a particular online activity was associated with PIU only in specific age groups, imply that particular age groups may be at risk of developing aspects of PIU. While young people might be more at risk of developing PIU with a propensity for viewing pornography, a vulnerability that may be less strong in middle age and wane later on in life, older people might be more prone to develop PIU characterized by problematic use of time wasters and streaming media (see exploratory Fig. 4). Finally, general surfing might be an underestimated facet of PIU, which seems to be more strongly associated with higher PIU scores in young people, but important across all age groups; this finding may be related to the fact that early adult life can be less goal directed and the young people are spending more time during unstructured activities in online environments compared to other older age groups.

Our results also have public health implications in relation to the regulation of online content, and targeting interventions. If particular activities are more strongly linked with the development of problematic use than others, then the question arises as to whether public health policies should be targeting groups of vulnerable individuals to improve their resilience towards risk of PIU, or whether more universal interventions targeting specific facets of internet behaviors, should be considered to make the online environments less addictive. For example, online platforms may in some cases be using specific architectures that take advantage of users' vulnerabilities (i.e. impulsive or compulsive traits) and that aim to maximize users' length of stay within the online environment. While this makes sense from a marketing perspective, it does raise concern of whether these environments should also issue a health warning to the user.

### 4.1 Limitations

This was a cross-sectional online survey, therefore no causal relationships can be drawn. Moreover, because of the recruitment methodology, and possible propensity for people with PIU to be more likely to complete an online survey, the current findings may not generalize to PIU in the general background population at large. Another limitation of our study is the lack of clinical data for some diagnostic entities associated with PIU, for example depression or substance misuse. Therefore, it is possible that depression or substance misuse could account for some of the associations observed in our study. Future studies should include a wider range of clinical parameters to explore whether those account for associations observed between PIU and internet activities. There are further limitations in regards to our clinical data deriving from using the MINI; this is validated to be delivered from a trained person in a face-to-face interview whereas in our study it was delivered via an online tool. However, our clinical data are in agreement with previous studies in PIU. Furthermore, another downside to our data collection, was that we assessed internet activity using time spent on the activity as proxy measure for PIU of that activity. While this can capture excessive, and therefore problematic use, it can also possibly capture essential use. While the activities assessed in this study were often by default non-essential due to their nature (e.g. time wasters), or when they are performed in severe excess (e.g. >8 h/day of shopping, gambling or pornography), future studies could include measures that can differentiate essential from non-essential internet use for each internet activity, to allow for such analyses. Another limitation of our study is the lack of data for children and adolescent populations. Children and adolescent populations may interact with the internet in a different way, but also are exposed to online use during a different neurodevelopmental window. Therefore, such differences may imply different vulnerabilities or resilience in terms of risk to develop PIU. For example, early, low level exposure to the online environment may have a ‘stress inoculation’ effect (Rutter, 1993) that steels individuals from future development of PIU. If such the case, this can further explain why older populations that only got their first exposure of online environments in adulthood may be more vulnerable. Future studies could include those child and adolescent age groups and examine prospectively whether specific internet activities are predictive of PIU. Unfortunately, the number of participants reporting transgender gender was small (n = 18), which did not allow for a meaningful analysis of the effect of transgender gender. A final limitation of our study is that our study population consists of healthy adults who only in <1% suffer from significant PIU behaviors (IAT > 80). Future studies would benefit from having a specific focus on the higher end of the PIU spectrum to be able to compare those severe PIU populations with a control group of low to moderate or non-PIU individuals. While the estimated point prevalence of PIU in our sample was ~8.5% (using IAT ≥ 50 cut-off), the thresholds for clinical caseness for PIU remain contentious and future research would benefit from a universally accepted measure and definition of PIU.

### 4.2 Conclusion

To summarize, DSM-5 highlights internet gaming disorder as a candidate disorder, but other types of online behavior (e.g. shopping, pornography, general surfing) bear a stronger relationship with maladaptive use of the internet than gaming. Psychiatric diagnoses and internet activities associated with Problematic internet use vary with age, a finding that has public health implications. These results contribute to the limited knowledge about internet activities associated with problematic internet use and may contribute to the diagnostic classification of problematic internet use as a multifaceted disorder.

## Role of funding sources

This research received internal departmental funds of the Department of Psychiatry at the University of Chicago. Dr. Ioannidis research activities are supported by Health Education East of England Higher Training Special interest sessions. Authors received no funding for the preparation of this manuscript. The funding source played no role in the design, data analysis, or writing of the study.

## Contributors

KI designed the idea for the manuscript, analysed the data, wrote the majority of the manuscript and Supplementary material and coordinated the co-authors' contributions. MT and FK participated in the conception and review of the statistical analysis. SRC, SR, DJS, CL and JEG designed and coordinated the study and collected and managed the data. All authors read and approved the final manuscript and contributed to the drafting and revising of the paper as well as to interpreting the results.

## Conflict of interest

Dr. Grant has received research grants from NIDA (RC1DA028279-01), the National Center for Responsible Gaming, and Roche and Forest Pharmaceuticals. Dr. Grant receives compensation from Springer as the editor-in-chief of the Journal of Gambling Studies and has received royalties from McGraw Hill, Oxford University Press, Norton, and the APPI. Dr. Chamberlain consults for Cambridge Cognition and his involvement in this research was supported by an Intermediate Clinical Fellowship from the Wellcome Trust (UK; 110049/Z/15/Z). Dan Stein and Christine Lochner are funded by the Medical Research Council of South Africa. The other authors report no financial relationships with commercial interest. None of the aforementioned sources had any role in the study design, collection, analysis or interpretation of the data, writing the manuscript, or the decision to submit the paper for publication.

## Appendix A Supplementary data

Supplementary material Image 1
