# Supplementary material for: Problematic internet use as an age-related multifaceted problem: Evidence from a two-site survey
Source: Addict Behav. 2018 Jun;81:157–66. doi: 10.1016/j.addbeh.2018.02.017 (PMC5849299; doi:10.1016/j.addbeh.2018.02.017)
Supplement: Supplementary file 1 — Supplementary material [file mmc1.pdf]

Methodology Appendix

Data variables and scores were loaded into the R-Studio workspace from the original database. Compound variables derive from combining values, or bins of values to make a new variable. These compound variables are readily available from well-established factor analyses in the common questionnaires like the Barratt Impulsivity Scale (BIS) and the Padua Inventory (PI). In our analysis we calculated total BIS and PADUA scores from adding the relevant subscales. Other continuous variables we used were age and internet activities scored on a zero to five integer scale referring to time spent per day over an activity (0 = None, 1 = <1 hr, 2 = 1-3 hrs, 3 = 4-5 hrs, 4 = 6-8 hrs, 5 = 8hrs<). All continuous variables including BIS, PI questionnaires, internet activities and Age were pre-processed (standardized) by subtracting the mean and dividing by the standard deviation. When comparing different models, pre-processing was included in the cross-validation process, so that each model would use the exact set of standardized scores to train and test predictions.

Binary and multi-level factor variables were used as originally coded: for Gender (1 = Male, 2 = Female), Race (1 = Caucasian, 2 = non-Caucasian, self-reported and then further categorized as a binary variable by the researchers), Education (1=<High School, 2=High School Graduates/General Education 3=Some College, 4=College Graduates, 5=Beyond College), Sexual Orientation (1 = Heterosexual, 2 = non-heterosexual, self-reported and then further categorized as a binary variable by the researchers), Relationship status (1=Single, 2=Married, 3=Divorced/Separated, 4=Living Together/Engaged, 5=Same sex relationship, 6=widow/er), Diagnostic variables for Attention deficit hyperactivity disorder (ADHD), Obsessive compulsive disorder (OCD), Generalized anxiety disorder (GAD) and Social anxiety disorder (SAD) were used as factor variables (1 = diagnosis is present, 0 = diagnosis is absent). Diagnosis for ADHD was assessed with the Adult ADHD self-reporting scale (ASRSv1.1) and the diagnoses for OCD, GAD and SAD were assessed with the MINI instrument. Interaction terms were created by simple algebraic multiplication of the two variables i.e. (Age * Internet activity) and (Gender * Internet activity).

To compare models and make predictions on Internet Addiction test scores (IAT scores), we created a data frame including all 51 predictor variables including 6 demographic variables (one continuous (Age) and five factor variables), two behaviour variables (BIS and PADUA), four clinical/diagnostic variables, 13 internet activity variables and 26 interaction variables. From hereon we will use the term DF to describe this data frame. We wanted to compare the various regularization and regression methods against linear regression and against a naïve baseline of prediction. For this purpose we used cross-validation to get out-of-sample estimates of RMSE for each of these models: Ridge regression, Elastic net, Lasso, Linear regression, Random forest and naïve baseline (i.e. predicting the average IAT score). This cross validation process was done in R environment using the caret package. The cross validation for ridge regression included linear regression (for $\lambda = 0$). We created a loop of 50 repeats where in each repeat DF was split randomly in a training (90%) and testing (10%) sets. A random seed was placed to allow replication of results. Each training set and each testing set had their continuous variables standardized separately. Models for each of the five methods were trained in the training set and then predictions were made in the test sets. The RMSE was calculated manually and stored in a data frame for later use. At the end of the 50 repeats the RMSE scores for each model were compared head to head using Exact Wilcoxon-Pratt signed rank test with Bonferroni correction for multiple comparisons.

Table S1a - Summary statistics for all variables for full data and stratified by age

Parameter	All (n= 1749)	18 ≤ Age ≤ 25 (n = 1042)	26 ≤ Age ≤ 55 (n = 592)	Age > 55 (n= 115)
**IAT score**	32.49[a](20-94)[b]	31.17[a](20-94)[b]	34.81[a](20-85)[b]	32.59 [a](20-72)[b]
**Age**	29(18-77)	20.8(18-25)	36.7(26-55)	61.9(56-77)
**Gender [male]**	1119[64]%	673[65]%	376[64]%	70[61]%
**Race [Caucasian]**	1285 [73]%	761[73]%	425[72]%	99[86]%
**Education [1=<High School, 2=High School Graduates/General Education 3=Some College, 4=College Graduates, 5=Beyond College]**	1:13<br>2:204<br>3:412<br>4:726<br>5:394	1:7<br>2:156<br>3:238<br>4:504<br>5:137	1:5<br>2:41<br>3:142<br>4:191<br>5:213	1:1<br>2:7<br>3:32<br>4:31<br>5:44
**Relationship status [1=Single, 2=Married, 3=Divorced/Separated, 4=Living Together/Engaged, 5=Same sex relationship, 6=widow/er]**	1:901<br>2:320<br>3:88<br>4:433<br>5:0<br>6:7	1:687<br>2:31<br>3:1<br>4:323<br>5:0<br>6:0	1:193<br>2:239<br>3:50<br>4:108<br>5:0<br>6:2	1:21<br>2:50<br>3:37<br>4:2<br>5:0<br>6:5
**Sexual orientation [heterosexual]**	1539 [88]%	930[89]%	509[86]%	100[87]%
**General Surfing**	1.87(0-5)	1.68(0-5)	2.10(0-5)	2.09(0-5)
**Internet gaming**	0.72(0-5)	0.61(0-5)	0.91(0-5)	0.77(0-5)
**RPG**	0.33(0-5)	0.33(0-5)	0.37(0-5)	0.10(0-4)
**Time Wasters**	0.83(0-5)	0.79(0-5)	0.91(0-5)	0.71(0-5)
**Action Multiplayer**	0.35(0-5)	0.35(0-5)	0.41(0-5)	0.13(0-5)
**Shopping**	0.79(0-5)	0.50(0-5)	1.21(0-5)	1.27(0-4)
**Auction Websites**	0.38(0-5)	0.21(0-5)	0.64(0-5)	0.60(0-5)
**Gambling**	0.09(0-5)	0.04(0-5)	0.18(0-5)	0.10(0-2)
**Social networking**	1.63(0-5)	1.58(0-5)	1.78(0-5)	1.29(0-5)
**Sports**	0.31(0-5)	0.22(0-5)	0.49(0-5)	0.27(0-2)
**Pornography**	0.51(0-5)	0.45(0-5)	0.64(0-5)	0.44(0-4)

**Messaging**	0.85(0-5)	0.85(0-5)	0.90(0-5)	0.60(0-5)
**Streaming media**	1.35(0-5)	1.33(0-5)	1.45(0-5)	0.99(0-4)
**PADUA**	29.2(0-136)	32.9(0-136)	24.9(0-131)	17.8(0-115)
**BIS**	63.5(35-120)	62.9(35-101)	64.9(35-120)	62.3(38-98)
**ADHD Diagnosis**	806 [46]%	519[50]%	252[43]%	35[30]%
**GAD Diagnosis**	363 [21]%	213[20]%	128[22]%	22[19]%
**Social Anxiety Diagnosis**	250 [14]%	163[16]%	82[14]%	5[4]%
**OCD Diagnosis**	191 [11]%	124[12]%	60[10]%	7[6]%

[a] mean; [b] range ; RPG - Role Playing games; PADUA: Padua Inventory-Revised Checking; BIS - Barratt Impulsiveness Scale 11; ADHD - Attention Deficit Hyperactivity Disorder; GAD – Generalized Anxiety disorder; OCD – Obsessive-Compulsive disorder; Chicago – USA subsample; Stellenbosch – South Africa subsample

Table S1b - Summary statistics for all variables for full data and stratified by site

Parameter	All (n= 1749)	Chicago<br>(n= 686)	Stellenbosch<br>(n= 1063)	p-value (Chicago vs Stellenbosch)	Effect size
**IAT score**	32.49[a](20-94)[b]	35.9 [a](20-85)[b]	30.3 [a](20-94)[b]	<0.05	0.06[c]
**Age**	29(18-77)	36.3(18-77)	24.3(18-76)	<0.05	0.20
**Gender [male]**	1119[64]%	503[73]%	616[58]%	<0.05	0.15[d]
**Race [Caucasian]**	1285 [73]%	518[76]%	767[72]%	n.s.	-
**Education**<br>**[1=<High School, 2=High School Graduates/General Education 3=Some College, 4=College Graduates, 5=Beyond College]**	1:13<br>2:204<br>3:412<br>4:726<br>5:394	1:12<br>2:60<br>3:228<br>4:250<br>5:136	1:1<br>2:144<br>3:184<br>4:476<br>5:258	n.s.	-
**Relationship status**<br>**[1=Single, 2=Married, 3=Divorced/Separated, 4=Living Together/Engaged, 5=Same sex relationship, 6=widow/er]**	1:901<br>2:320<br>3:88<br>4:433<br>5:0<br>6:7	1:269<br>2:194<br>3:76<br>4:143<br>5:0<br>6:4	1:632<br>2:216<br>3:12<br>4:290<br>5:0<br>6:3	n.s.	-
**Sexual orientation [heterosexual]**	1539 [88]%	575[84]%	964[91]%	<0.05	0.10
**General Surfing**	1.87(0-5)	2.32(0-5)	1.57(0-5)	<0.05	0.11
**Internet gaming**	0.72(0-5)	0.99(0-5)	0.54(0-5)	<0.05	0.04
**RPG**	0.33(0-5)	0.38(0-5)	0.30(0-5)	n.s.	-
**Time Wasters**	0.83(0-5)	0.93(0-5)	0.76(0-5)	<0.05	0.01
**Action Multiplayer**	0.35(0-5)	0.36(0-5)	0.35(0-5)	n.s.	-
**Shopping**	0.79(0-5)	1.27(0-5)	0.48(0-5)	<0.05	0.18
**Auction Websites**	0.38(0-5)	0.63(0-5)	0.22(0-5)	<0.05	0.07
**Gambling**	0.09(0-5)	0.13(0-5)	0.64(0-5)	0.03	<0.01
**Social networking**	1.63(0-5)	1.81(0-5)	1.51(0-5)	<0.05	0.02
**Sports**	0.31(0-5)	0.39(0-5)	0.26(0-5)	<0.05	<0.01
**Pornography**	0.51(0-5)	0.54(0-5)	0.49(0-5)	n.s.	-
**Messaging**	0.85(0-5)	0.88(0-5)	0.82(0-5)	n.s.	-
**Streaming media**	1.35(0-5)	1.6(0-5)	1.2(0-5)	<0.05	0.04
**PADUA**	29.2(0-136)	22.9(0-136)	33.3(0-134)	<0.05	0.05
**BIS**	63.5(35-120)	63.7(35-99)	63.4(35-120)	n.s.	-
**ADHD Diagnosis**	806 [46]%	279[41]%	527[50]%	<0.05	0.09
**GAD Diagnosis**	363 [21]%	164[24]%	199[19]%	n.s.	-

**Social Anxiety Diagnosis**	250 [14]%	97[14]%	153[14]%	n.s.	-
**OCD Diagnosis**	191 [11]%	62[9]%	129[12]%	n.s.	-

[a] mean; [b] range ; [c] partial eta-square; [d] phi(φ); comparison statistics between the Stellenbosch and the Chicago sample are ANOVA and partial eta squares (effect size) for the continuous variables and chi square and phi for the categorical variables. P-values are corrected with Bonferroni correction and effect sizes of non-significant values are not reported. RPG - Role Playing games; PADUA: Padua Inventory-Revised Checking; BIS - Barratt Impulsiveness Scale 11; ADHD - Attention Deficit Hyperactivity Disorder; GAD – Generalized Anxiety disorder; OCD – Obsessive-Compulsive disorder; Chicago – USA subsample; Stellenbosch – South Africa subsample; n.s. – non-significant

Table S2 - Model comparisons: Linear regression, Ridge, elastic net, random forest and LASSO

	LR	Ridge	Elastic Net	Lasso	Random Forest	Naive Baseline
**LR**		**Effect size d = 0.78**	**Effect size d = 0.76**	**Effect size d = 0.76**	**Effect size d = 0.44**	**Effect size d = -0.87**
**Ridge**	**p < .001 (corrected <.001)**		**Effect size d = 0.49**	**Effect size d = 0.51**	**Effect size d = 0.002**	**Effect size d = -0.87**
**Elastic Net**	**P < .001 (corrected <.001)**	**P < .001 (corrected =0.006)**		**Effect size d = -0.08**	**Effect size d = -0.27**	**Effect size d = -0.87**
**Lasso**	**p < .001 (corrected <.001)**	**p < .001 (p-corrected = 0.003)**	**Equal p = 0.60**		**Effect size d = -0.21**	**Effect size d = -0.87**
**Random Forest**	**p = 0.0013 (corrected <.05)**	**Equal p = 0.99**	**Equal p = 0.048 (corrected >.05)**	**Equal p = 0.12**		**Effect size d = -0.87**
**Naïve Baseline**	**p < .001 (corrected <.001)**	**p < .001 (corrected <.001)**	**P < .001 (corrected <.001)**	**p < .001 (corrected <.001)**	**p<.001 (corrected <.001)**	

LR = linear regression models; E.net = elastic net models; Ridge = Ridge regression models; Lasso = least absolute shrinkage and selection operator (regression) models. All models are compared using cross-validated, out-of-sample root mean squared error (RMSE) as comparative metric. P values are Exact Wilcoxon-Pratt signed rank test (column better than row, one-sided. Effect sizes of Cohen’s d (upper half); Significances of p-values (lower half) with Bonferroni correction (*18) for multiple comparisons.

Table S3 – Summary statistics for RMSE scores for different models

	Median	Standard deviation	min	max
**LR**	8.13	0.281	7.35	8.72
**Lasso**	8.01	0.281	7.19	8.60
**Elastic Net**	8.00	0.280	7.12	8.63
**Ridge regression**	8.05	0.267	7.24	8.56
**Random Forest**	8.04	0.276	7.32	8.55
**Naive Baseline**	10.80	0.364	9.86	11.40

N= 50 for all models; LR = linear regression models; E.net = elastic net models; Ridge = Ridge regression models; Lasso = least absolute shrinkage and selection operator (regression) models. All models are compared using cross-validated, out-of-sample root mean squared error (RMSE) as comparative metric

Table S4 – Linear regression and lasso coefficients for the full data with interaction (full results)

	Estimate	Std. Error	t value	Pr(>|t|)	LASSO
**Age**	0.8800	0.460	1.900	0.06	0.000
**Gender**	1.8000	0.950	1.900	0.06	0.000
**Race**	0.9900	0.450	2.200	0.03	0.000
**Relationship status (5 levels)**	0.4700<br>0.5900<br>-0.5300<br>-2.1000	0.610<br>1.100<br>0.470<br>3.100	0.770<br>0.560<br>-1.100<br>-0.670	0.44<br>0.58<br>0.26<br>0.5	0.000<br>0.000<br>0.000<br>0.000
**Education (5 levels)**	-1.1000<br>-0.2900<br>-0.3600<br>-0.3900	2.300<br>2.200<br>2.200<br>2.200	-0.470<br>-0.130<br>-0.160<br>-0.170	0.64<br>0.9<br>0.87<br>0.86	0.000<br>0.000<br>0.000<br>0.000
**Sexual Orientation**	1.5000	0.64	2.4000	0.02	0.000
**General Surfing**	4.3000	0.950	4.500	< 0.001	**2.100**
**Internet gaming**	0.6600	1.200	0.560	0.58	**0.600**
**RPG**	-1.2000	1.000	-1.200	0.23	0.000
**Time Wasters**	0.1100	0.860	0.130	0.9	0.000
**Action Multiplayer**	0.5300	1.100	0.490	0.63	0.000
**Shopping**	1.5000	0.990	1.600	0.12	**1.400**
**Auction Websites**	1.2000	1.000	1.100	0.26	**0.027**
**Gambling**	-0.5100	0.980	-0.510	0.61	0.000
**Social networking**	2.6000	0.850	3.100	< 0.01	**0.460**
**Sports**	-0.2800	0.970	-0.290	0.77	0.000
**Pornography**	1.4000	0.980	1.400	0.16	**1.000**
**Messaging**	-0.8300	0.770	-1.100	0.28	0.000
**Streaming media**	-1.1000	0.900	-1.200	0.23	0.000
**Age x General Surfing**	-2.0000	0.760	-2.700	< 0.01	0.000
**Age x Internet Gaming**	-0.7800	0.720	-1.100	0.28	0.000
**Age x RPG**	1.6000	0.660	2.400	0.02	**0.330**
**Age x Time Wasters**	0.9200	0.590	1.500	0.12	0.000
**Age x Action Multiplayer**	-0.0670	0.690	-0.098	0.92	0.000

**Age x Shopping**	-0.6500	0.790	-0.820	0.41	0.000
**Age x Gambling**	0.7000	0.700	1.000	0.31	**0.150**
**Age x Auction Websites**	1.1000	0.730	1.500	0.14	**0.350**
**Age x Social Networking**	0.0073	0.650	0.011	0.99	0.000
**Age x Sports**	1.0000	0.630	1.600	0.11	0.000
**Age x Pornography**	-1.3000	0.630	-2.000	0.04	0.000
**Age x Messaging**	0.8100	0.580	1.400	0.16	0.000
**Age x Streaming media**	0.5400	0.660	0.820	0.41	**0.350**
**Gender x General Surfing**	-0.8300	0.820	-1.000	0.31	0.000
**Gender x Internet Gaming**	0.9900	0.910	1.100	0.28	0.000
**Gender x RPG**	0.1200	0.820	0.150	0.88	0.000
**Gender x Time Wasters**	-0.9000	0.690	-1.300	0.19	0.000
**Gender x Action Multiplayer**	-0.4900	0.930	-0.530	0.6	0.000
**Gender x Shopping**	0.4100	0.800	0.510	0.61	0.000
**Gender x Gambling**	0.3700	0.780	0.470	0.64	0.000
**Gender x Auction Websites**	-1.7000	0.770	-2.200	0.03	0.000
**Gender x Social Networking**	-2.0000	0.750	-2.700	< 0.01	0.000
**Gender x Sports**	-0.5900	0.830	-0.700	0.48	0.000
**Gender x Pornography**	1.1000	0.900	1.300	0.21	0.000
**Gender x Messaging**	0.1500	0.650	0.230	0.82	0.000
**Gender x Streaming media**	1.1000	0.780	1.500	0.14	0.000
**PADUA**	0.0870	0.018	4.800	< 0.001	**0.074**
**BIS**	0.0780	0.010	7.800	< 0.001	**0.066**
**ADHD Diagnosis**	2.9000	0.420	7.000	< 0.001	**1.700**
**OCD Diagnosis**	1.5000	0.680	2.200	0.03	**0.230**
**Social Anxiety Diagnosis**	0.8000	0.620	1.300	0.19	0.000
**GAD Diagnosis**	1.0000	0.540	1.900	0.06	**0.270**

T value - coefficient of linear regression; Pr(>|t|) - p-value of linear regression; Lasso - least absolute shrinkage and selection operator; RPG - Role Playing games; PADUA: Padua Inventory-Revised Checking; BIS - Barratt Impulsiveness Scale 11; ADHD - Attention Deficit Hyperactivity Disorder; GAD – Generalized Anxiety disorder; OCD – Obsessive-Compulsive disorder;

Linear regression statistics: Residual standard error: 7.812 on 1691 degrees of freedom, Multiple R-squared: 0.4992, Adjusted R-squared: 0.4823, F-statistic: 29.57 on 57 and 1691 DF, p-value: < 2.2e-16

Table S5 - Subgroup analyses – Young people (ages 18-25, 1042 participants)

	Estimate	Std. Error	t value	Pr(>|t|)	LASSO
**Gender**	0.590	1.300	0.430	0.66	0.000
**Race**	0.820	0.570	1.400	0.15	0.000
**Relationship status (4 levels)**	1.900<br>16.000<br>-1.000	1.500<br>7.800<br>0.540	1.300<br>2.000<br>-1.900	0.21<br>0.05<br>0.06	0.000<br>0.000<br>0.000
**Education (5 levels)**	-0.630<br>-0.550<br>-0.770<br>-1.200	3.100<br>3.000<br>3.000<br>3.100	-0.210<br>-0.180<br>-0.250<br>-0.390	0.84<br>0.86<br>0.8<br>0.7	0.000<br>0.000<br>0.000<br>0.000
**Sexual Orientation**	2.200	0.850	2.500	0.01	0.000
**General Surfing**	2.900	0.910	3.200	< 0.01	**2.400**
**Internet gaming**	-0.260	1.200	-0.220	0.83	**0.450**
**RPG**	1.400	1.000	1.300	0.18	0.000
**Time Wasters**	0.770	0.820	0.940	0.35	0.000
**Action Multiplayer**	1.200	1.100	1.100	0.28	0.000
**Shopping**	0.910	0.930	0.980	0.33	**0.840**
**Auction Websites**	0.350	0.920	0.380	0.7	0.000
**Gambling**	-1.100	0.980	-1.100	0.26	0.000
**Social networking**	1.600	0.860	1.900	0.06	0.000
**Sports**	-0.100	1.000	-0.098	0.92	0.000
**Pornography**	0.900	1.000	0.860	0.39	**1.400**
**Messaging**	-1.000	0.780	-1.300	0.2	0.000
**Streaming media**	-0.570	0.850	-0.670	0.5	0.000
**Gender x General Surfing**	-0.650	1.000	-0.640	0.52	0.000
**Gender x Internet Gaming**	1.700	1.200	1.400	0.16	0.000
**Gender x RPG**	-1.800	1.100	-1.700	0.1	0.000
**Gender x Time Wasters**	-0.970	0.850	-1.100	0.25	0.000
**Gender x Action Multiplayer**	-1.000	1.100	-0.900	0.37	0.000
**Gender x Shopping**	0.370	0.960	0.380	0.7	0.000

**Gender x Gambling**	0.640	1.000	0.630	0.53	0.000
**Gender x Auction Websites**	-0.210	0.980	-0.220	0.83	0.000
**Gender x Social Networking**	-1.200	0.920	-1.400	0.18	0.000
**Gender x Sports**	-0.058	1.100	-0.054	0.96	0.000
**Gender x Pornography**	0.980	1.100	0.870	0.38	0.000
**Gender x Messaging**	0.760	0.810	0.930	0.35	0.000
**Gender x Streaming media**	0.990	0.980	1.000	0.31	0.000
**PADUA**	0.100	0.024	4.300	< 0.001	**0.085**
**BIS**	0.067	0.013	5.300	< 0.001	**0.048**
**ADHD Diagnosis**	2.100	0.530	3.900	< 0.001	**0.350**
**OCD Diagnosis**	0.990	0.820	1.200	0.22	0.000
**Social Anxiety Diagnosis**	2.100	0.750	2.700	< 0.01	**0.560**
**GAD Diagnosis**	0.290	0.700	0.420	0.68	0.000

T value - coefficient of linear regression; Pr(>|t|) - p-value of linear regression; Lasso - least absolute shrinkage and selection operator; RPG - Role Playing games; PADUA: Padua Inventory-Revised Checking; BIS - Barratt Impulsiveness Scale 11; ADHD - Attention Deficit Hyperactivity Disorder; GAD – Generalized Anxiety disorder; OCD – Obsessive-Compulsive disorder; Linear regression statistics: Residual standard error: 7.686 on 999 degrees of freedom; Multiple R-squared: 0.4404, Adjusted R-squared: 0.4169 ; F-statistic: 18.72 on 42 and 999 DF, p-value: < 2.2e-16

Table S6 - Subgroup analyses – Middle aged people (ages 26-55, 592 participants)

	Estimate	Std. Error	t value	Pr(>|t|)	LASSO
**Gender**	3.300	1.700	2.000	0.05	0.000
**Race**	1.300	0.800	1.700	0.1	0.000
**Relationship status (5 levels)**	0.400<br>0.110<br>0.670<br>-1.800	0.820<br>1.400<br>0.990<br>6.000	0.480<br>0.079<br>0.670<br>-0.310	0.63<br>0.94<br>0.5<br>0.76	0.000<br>0.000<br>0.000<br>0.000
**Education (5 levels)**	-4.000<br>-0.870<br>-1.200<br>-1.600	3.900<br>3.700<br>3.700<br>3.700	-1.000<br>-0.240<br>-0.330<br>-0.440	0.31<br>0.81<br>0.74<br>0.66	0.000<br>0.000<br>0.000<br>0.000
**Sexual Orientation**	-0.440	1.000	-0.420	0.67	0.000
**General Surfing**	1.500	1.300	1.100	0.27	**1.500**
**Internet gaming**	0.730	1.500	0.490	0.62	**0.110**
**RPG**	-1.500	1.500	-1.000	0.31	**0.710**
**Time Wasters**	0.660	1.300	0.510	0.61	0.000
**Action Multiplayer**	0.650	1.700	0.390	0.7	0.000
**Shopping**	0.520	1.300	0.390	0.7	**1.500**
**Auction Websites**	3.600	1.300	2.700	< 0.01	**0.990**
**Gambling**	0.540	1.500	0.370	0.71	**0.780**
**Social networking**	5.100	1.300	3.800	< 0.001	**1.300**
**Sports**	1.000	1.400	0.750	0.46	0.000
**Pornography**	-0.240	1.500	-0.170	0.87	**0.210**
**Messaging**	0.970	1.200	0.780	0.44	**0.110**
**Streaming media**	-0.780	1.300	-0.600	0.55	0.000
**Gender x General Surfing**	0.430	1.600	0.270	0.78	0.000
**Gender x Internet Gaming**	-0.170	1.500	-0.110	0.91	0.000
**Gender x RPG**	2.900	1.500	2.000	0.05	0.000
**Gender x Time Wasters**	-0.470	1.300	-0.350	0.72	0.000
**Gender x Action Multiplayer**	-1.700	1.800	-0.990	0.32	0.000

**Gender x Shopping**	1.200	1.400	0.830	0.41	0.000
**Gender x Gambling**	1.100	1.500	0.750	0.46	0.000
**Gender x Auction Websites**	-2.800	1.300	-2.100	0.04	0.000
**Gender x Social Networking**	-4.100	1.500	-2.800	< 0.01	0.000
**Gender x Sports**	-1.100	1.500	-0.710	0.48	0.000
**Gender x Pornography**	1.300	1.700	0.790	0.43	0.000
**Gender x Messaging**	-0.580	1.300	-0.450	0.65	0.000
**Gender x Streaming media**	0.900	1.500	0.620	0.54	0.000
**PADUA**	0.054	0.032	1.700	0.1	**0.029**
**BIS**	0.085	0.018	4.600	< 0.001	**0.072**
**ADHD Diagnosis**	4.800	0.760	6.400	< 0.001	**3.100**
**OCD Diagnosis**	1.800	1.300	1.300	0.18	0.000
**Social Anxiety Diagnosis**	-2.000	1.200	-1.700	0.09	0.000
**GAD Diagnosis**	1.700	0.950	1.800	0.07	0.000

T value - coefficient of linear regression; Pr(>|t|) - p-value of linear regression; Lasso - least absolute shrinkage and selection operator; RPG - Role Playing games; PADUA: Padua Inventory-Revised Checking; BIS - Barratt Impulsiveness Scale 11; ADHD - Attention Deficit Hyperactivity Disorder; GAD – Generalized Anxiety disorder; OCD – Obsessive-Compulsive disorder; Linear regression statistics: Residual standard error: 7.998 on 548 degrees of freedom ; Multiple R-squared: 0.5766, Adjusted R-squared: 0.5433 ; F-statistic: 17.35 on 43 and 548 DF, p-value: < 2.2e-16

Table S7 - Subgroup analyses - Older people (ages over 55, 115 participants)

	Estimate	Std. Error	t value	Pr(>|t|)	LASSO
**Gender**	0.0025	3.000	0.00083	1	0.000
**Race**	-0.1500	2.200	-0.06600	0.95	0.000
**Relationship status (5 levels)**	2.8000<br>4.5000<br>-1.3000<br>-0.2800	2.100<br>2.200<br>6.800<br>3.800	1.30000<br>2.00000<br>-0.19000<br>-0.07500	0.2<br>0.05<br>0.85<br>0.94	0.000<br>0.000<br>0.000<br>0.000
**Education (5 levels)**	1.5000<br>9.7000<br>10.0000<br>11.0000	7.600<br>7.100<br>7.100<br>7.100	0.19000<br>1.40000<br>1.40000<br>1.50000	0.85<br>0.18<br>0.16<br>0.13	0.000<br>0.000<br>0.000<br>0.000
**Sexual Orientation**	1.6000	2.500	0.66000	0.51	0.000
**General Surfing**	2.2000	2.600	0.85000	0.4	**0.590**
**Internet gaming**	-0.0570	3.300	-0.01700	0.99	0.000
**RPG**	2.1000	3.200	0.66000	0.51	0.000
**Time Wasters**	2.5000	2.800	0.91000	0.37	**0.450**
**Action Multiplayer**	-1.0000	12.000	-0.08600	0.93	0.000
**Shopping**	0.3600	2.700	0.13000	0.9	0.000
**Auction Websites**	0.3400	3.300	0.10000	0.92	**0.230**
**Gambling**	4.8000	2.500	2.00000	0.05	0.000
**Social networking**	0.8800	3.000	0.29000	0.77	0.000
**Sports**	0.2100	3.300	0.06300	0.95	0.000
**Pornography**	0.9400	5.400	0.17000	0.86	0.000
**Messaging**	-0.3400	2.800	-0.12000	0.9	0.000
**Streaming media**	-0.3700	2.800	-0.13000	0.9	**1.200**
**Gender x General Surfing**	-0.3800	3.000	-0.13000	0.9	0.000
**Gender x Internet Gaming**	1.2000	4.100	0.30000	0.76	0.000
**Gender x RPG**	-1.6000	3.600	-0.46000	0.65	0.000
**Gender x Time Wasters**	-1.7000	2.700	-0.66000	0.51	0.000
**Gender x Action Multiplayer**	2.3000	12.000	0.19000	0.85	0.000

**Gender x Shopping**	-1.5000	2.800	-0.55000	0.59	0.000
**Gender x Gambling**	-3.8000	2.500	-1.50000	0.13	0.000
**Gender x Auction Websites**	0.8400	3.300	0.26000	0.8	0.000
**Gender x Social Networking**	-0.6400	3.000	-0.22000	0.83	0.000
**Gender x Sports**	0.2600	3.500	0.07500	0.94	0.000
**Gender x Pornography**	-1.6000	5.700	-0.28000	0.78	0.000
**Gender x Messaging**	-0.9900	3.000	-0.33000	0.74	0.000
**Gender x Streaming media**	2.9000	3.100	0.93000	0.36	0.000
**PADUA**	0.2000	0.068	3.00000	< 0.01	**0.065**
**BIS**	0.0860	0.052	1.70000	0.1	**0.086**
**ADHD Diagnosis**	-1.2000	1.800	-0.67000	0.51	0.000
**OCD Diagnosis**	13.0000	3.800	3.40000	< 0.01	**6.400**
**Social Anxiety Diagnosis**	5.8000	4.200	1.40000	0.17	0.000
**GAD Diagnosis**	2.6000	2.300	1.10000	0.27	**4.300**

T value - coefficient of linear regression; Pr(>|t|) - p-value of linear regression; Lasso - least absolute shrinkage and selection operator; RPG - Role Playing games; PADUA: Padua Inventory-Revised Checking; BIS - Barratt Impulsiveness Scale 11; ADHD - Attention Deficit Hyperactivity Disorder; GAD – Generalized Anxiety disorder; OCD – Obsessive-Compulsive disorder; Linear regression statistics: Residual standard error: 6.214 on 71 degrees of freedom; Multiple R-squared: 0.7859, Adjusted R-squared: 0.6562 ; F-statistic: 6.06 on 43 and 71 DF, p-value: 1.517e-11

Table S8 - Subgroup analyses – Stratified by study site (Chicago (CHI) and Stellenbosch (SA))

	Estimate (CHI)	Std. Error	Pr(>|t|) (CHI)	LASSO (CHI)	Estimate (SA)	Std. Error	Pr(>|t|) (SA)	LASSO (SA)
**Gender**	1.9000	1.300	0.14	0.0000	1.700	1.200	0.16	0.0000
**Age**	-0.6900	0.490	0.16	0.0000	-0.440	0.770	0.57	0.0000
**Race**	1.1000	0.750	0.16	0.0000	0.820	0.540	0.13	0.0000
**Relationship**	-0.3600	0.810	0.66	0.0000	1.800	0.980	0.07	0.0000
	-0.2400	1.200	0.84	0.0000	2.000	2.600	0.45	0.0000
	0.4500	0.840	0.59	0.0000	-0.680	0.540	0.21	0.0000
	-1.9000	4.100	0.65	0.0000	0.130	5.200	0.98	0.0000
**Sexual Orientation**	-1.5000	0.890	0.1	0.0000	3.200	0.870	< 0.001	**0.6500**
**Education**	-0.3000	2.600	0.91	0.0000	3.900	7.700	0.61	0.0000
	-0.1700	2.400	0.94	0.0000	5.100	7.700	0.51	0.0000
	1.5000	2.400	0.53	0.0000	4.300	7.700	0.58	0.0000
	2.4000	2.400	0.33	0.0000	3.900	7.700	0.62	0.0000
**General Surfing**	2.2000	1.100	0.05	**0.5400**	4.400	2.300	0.06	**4.5000**
**Internet gaming**	-0.8100	1.500	0.59	**0.0820**	3.500	2.300	0.13	**0.3300**
**RPG**	-0.5000	1.400	0.73	**0.4400**	-0.410	1.800	0.82	0.0000
**Time Wasters**	0.0240	1.300	0.98	**0.1600**	1.900	1.500	0.2	0.0000
**Action Multiplayer**	0.4800	1.500	0.74	0.0000	-0.430	1.900	0.82	0.0000
**Shopping**	0.2100	1.300	0.88	**0.5200**	-2.500	1.800	0.16	**0.0540**
**Auction Websites**	2.9000	1.400	0.04	**0.1600**	-0.520	2.000	0.79	0.0000
**Gambling**	1.8000	1.300	0.18	**0.2400**	-5.900	1.700	< 0.001	0.0000
**Social networking**	1.7000	1.100	0.12	**0.7200**	4.700	1.800	0.01	**0.4200**
**Sports**	-0.8900	1.400	0.52	0.0000	0.590	1.500	0.7	0.0000
**Pornography**	-3.0000	1.400	0.03	0.0000	6.900	1.700	< 0.001	**2.3000**
**Messaging**	2.2000	1.100	0.05	**0.0620**	-3.600	1.400	0.01	0.0000
**Streaming media**	-0.9300	1.100	0.42	**0.0004**	-0.770	1.800	0.67	0.0000
**Age x General Surfing**	-0.3500	0.800	0.67	0.0000	-0.690	3.000	0.82	0.0000
**Age x Internet Gaming**	0.4500	0.880	0.61	0.0000	-3.900	2.400	0.1	**0.6100**
**Age x RPG**	0.6700	0.850	0.43	**0.0120**	1.500	1.800	0.38	0.0000
**Age x Time Wasters**	0.3100	0.790	0.69	0.0000	0.340	1.800	0.85	0.0000
**Age x Action Multiplayer**	-0.5000	0.880	0.57	0.0000	1.600	1.900	0.41	0.0000

**Age x Shopping**	-0.1100	0.880	0.9	0.0000	2.700	2.100	0.2	**1.5000**
**Age x Gambling**	-0.7300	1.100	0.51	0.0000	2.300	1.600	0.15	0.0000
**Age x Auction Websites**	0.7900	0.930	0.39	0.0000	1.200	1.800	0.5	**0.5500**
**Age x Social Networking**	-0.2700	0.800	0.74	0.0000	-1.200	2.100	0.58	0.0000
**Age x Sports**	0.5700	0.970	0.56	0.0000	1.500	1.100	0.18	0.0000
**Age x Pornography**	1.8000	0.870	0.04	0.0000	-3.800	1.600	0.02	0.0000
**Age x Messaging**	-0.5200	0.750	0.49	0.0000	4.300	1.600	< 0.01	0.0000
**Age x Streaming media**	0.3800	0.790	0.63	0.0000	-0.230	1.900	0.9	0.0000
**Gender x General Surfing**	-1.3000	0.900	0.16	0.0000	0.560	1.700	0.75	0.0000
**Gender x Internet Gaming**	0.9600	1.100	0.4	0.0000	0.630	1.700	0.71	0.0000
**Gender x RPG**	0.9400	1.100	0.38	0.0000	-0.980	1.300	0.46	0.0000
**Gender x Time Wasters**	-0.0083	1.000	0.99	0.0000	-2.300	1.000	0.03	0.0000
**Gender x Action Multiplayer**	0.0520	1.200	0.97	0.0000	-0.490	1.500	0.74	0.0000
**Gender x Shopping**	0.4800	1.100	0.67	0.0000	2.100	1.300	0.1	0.0000
**Gender x Gambling**	-0.1600	0.970	0.87	0.0000	3.500	1.400	0.02	0.0000
**Gender x Auction Websites**	-4.0000	0.980	< 0.001	0.0000	0.150	1.400	0.91	0.0000
**Gender x Social Networking**	-0.7500	0.910	0.41	0.0000	-2.800	1.500	0.06	0.0000
**Gender x Sports**	0.2400	1.200	0.84	0.0000	-1.900	1.300	0.15	0.0000
**Gender x Pornography**	1.6000	1.400	0.27	0.0000	-2.100	1.400	0.15	0.0000
**Gender x Messaging**	-1.4000	0.930	0.14	0.0000	0.550	1.000	0.58	0.0000
**Gender x Streaming media**	0.7200	0.900	0.42	0.0000	1.100	1.400	0.41	0.0000
**PADUA**	0.1800	0.033	< 0.001	**0.1400**	0.035	0.022	0.12	**0.0091**
**BIS**	0.0990	0.017	< 0.001	**0.0950**	0.081	0.012	< 0.001	**0.0670**
**ADHD Diagnosis**	2.8000	0.720	< 0.001	**1.7000**	2.900	0.500	< 0.001	**1.7000**
**GAD Diagnosis**	2.3000	1.200	0.06	**1.1000**	1.000	0.800	0.2	0.0000
**Social Anxiety Diagnosis**	-0.7200	1.000	0.49	0.0000	1.400	0.750	0.05	**0.1600**
**OCD Diagnosis**	2.5000	0.860	< 0.01	**1.0000**	-0.270	0.690	0.69	0.0000

T value - coefficient of linear regression; Pr(>|t|) - p-value of linear regression; Lasso - least absolute shrinkage and selection operator; RPG - Role Playing games; PADUA: Padua Inventory-Revised Checking; BIS - Barratt Impulsiveness Scale 11; ADHD - Attention Deficit Hyperactivity Disorder; GAD – Generalized Anxiety disorder; OCD – Obsessive-Compulsive disorder;

Table S9 - Intermediate models results – without impulsivity & compulsivity variables

	**Estimate**	**Std. Error**	**t value**	**Pr(>|t|)**	**LASSO**
**Gender**	1.4000	0.79	1.70000	0.08	0.00
**Age**	0.8200	0.39	2.10000	0.03	0.00
**Race**	1.5000	0.47	3.30000	< 0.01	0.00
**Relationship**	0.6100	0.64	0.95000	0.34	0.00
	0.7600	1.10	0.67000	0.5	0.00
	-0.7000	0.49	-1.40000	0.16	0.00
	-1.4000	3.30	-0.43000	0.67	0.00
**Sexual Orientation**	1.8000	0.65	2.70000	< 0.01	0.00
**Education**	-1.1000	2.40	-0.46000	0.65	0.00
	-0.3000	2.40	-0.13000	0.9	0.00
	-0.9900	2.40	-0.42000	0.68	0.00
	-1.4000	2.40	-0.57000	0.57	0.00
**General Surfing**	4.0000	1.00	3.90000	< 0.001	**1.70**
**Internet gaming**	-0.5900	1.30	-0.46000	0.64	**0.61**
**RPG**	-0.8900	1.10	-0.80000	0.43	0.00
**Time Wasters**	-0.0510	0.98	-0.05200	0.96	0.00
**Action Multiplayer**	1.5000	1.20	1.30000	0.2	0.00
**Shopping**	1.3000	1.10	1.20000	0.24	**1.30**
**Auction Websites**	1.4000	1.20	1.20000	0.22	**0.53**
**Gambling**	0.9200	1.00	0.89000	0.38	**0.16**
**Social networking**	2.8000	0.96	3.00000	< 0.01	**0.62**
**Sports**	-1.5000	1.10	-1.40000	0.16	0.00
**Pornography**	1.9000	1.00	1.80000	0.07	**1.20**
**Messaging**	-0.6500	0.86	-0.75000	0.45	0.00
**Streaming media**	-0.7700	0.99	-0.77000	0.44	0.00
**Age x General Surfing**	-1.9000	0.75	-2.50000	0.01	0.00
**Age x Internet Gaming**	-0.5600	0.78	-0.71000	0.48	0.00
**Age x RPG**	1.4000	0.71	1.90000	0.05	**0.34**
**Age x Time Wasters**	1.2000	0.65	1.90000	0.06	0.00
**Age x Action Multiplayer**	-0.3100	0.73	-0.42000	0.67	0.00
**Age x Shopping**	-1.4000	0.77	-1.80000	0.07	0.00

**Age x Gambling**	-0.1700	0.82	-0.20000	0.84	0.00
**Age x Auction Websites**	0.9400	0.81	1.20000	0.25	0.00
**Age x Social Networking**	-0.3200	0.72	-0.45000	0.65	0.00
**Age x Sports**	1.5000	0.71	2.20000	0.03	0.00
**Age x Pornography**	-0.9600	0.65	-1.50000	0.14	0.00
**Age x Messaging**	1.2000	0.63	1.90000	0.06	0.00
**Age x Streaming media**	0.3400	0.72	0.47000	0.64	**0.31**
**Gender x General Surfing**	-0.7700	0.80	-0.97000	0.33	0.00
**Gender x Internet Gaming**	2.0000	0.94	2.10000	0.04	0.00
**Gender x RPG**	0.0004	0.87	0.00047	1	0.00
**Gender x Time Wasters**	-0.9100	0.75	-1.20000	0.23	0.00
**Gender x Action Multiplayer**	-1.2000	0.98	-1.20000	0.22	0.00
**Gender x Shopping**	1.2000	0.83	1.50000	0.13	0.00
**Gender x Gambling**	-0.1700	0.82	-0.21000	0.84	0.00
**Gender x Auction Websites**	-1.8000	0.81	-2.20000	0.03	0.00
**Gender x Social Networking**	-1.7000	0.79	-2.10000	0.03	0.00
**Gender x Sports**	0.2500	0.89	0.28000	0.78	0.00
**Gender x Pornography**	0.3900	1.00	0.39000	0.7	0.00
**Gender x Messaging**	-0.4600	0.70	-0.66000	0.51	0.00
**Gender x Streaming media**	0.9000	0.78	1.20000	0.25	0.00
**ADHD Diagnosis**	4.1000	0.42	9.70000	< 0.001	**2.50**
**GAD Diagnosis**	2.0000	0.56	3.60000	< 0.001	**1.00**
**Social Anxiety Diagnosis**	1.8000	0.64	2.90000	< 0.01	**0.77**
**OCD Diagnosis**	2.5000	0.70	3.60000	< 0.001	**1.20**

T value - coefficient of linear regression; Pr(>|t|) - p-value of linear regression; Lasso - least absolute shrinkage and selection operator; RPG - Role Playing games; PADUA: Padua Inventory-Revised Checking; BIS - Barratt Impulsiveness Scale 11; ADHD - Attention Deficit Hyperactivity Disorder; GAD – Generalized Anxiety disorder; OCD – Obsessive-Compulsive disorder;

Table S10 - Intermediate models – without diagnostic variables

Variable	Estimate	Std. Error	t value	Pr(>|t|)	LASSO
**Gender**	1.7000	0.83	2.0000	0.05	0.000
**Age**	0.2800	0.41	0.6800	0.5	0.000
**Race**	1.1000	0.49	2.2000	0.03	0.000
**Relationship**	0.6500	0.68	0.9600	0.34	0.000
	1.0000	1.20	0.8700	0.38	0.000
	-0.8000	0.52	-1.5000	0.13	0.000
	-0.8600	3.50	-0.2500	0.8	0.000
**Sexual Orientation**	1.9000	0.69	2.8000	< 0.01	0.000
**Education**	-2.3000	2.60	-0.9100	0.37	0.000
	-1.2000	2.50	-0.4800	0.63	0.000
	-2.0000	2.50	-0.8100	0.42	0.000
	-2.6000	2.50	-1.0000	0.29	0.000
**General Surfing**	4.3000	1.10	4.0000	< 0.001	**1.700**
**Internet gaming**	-0.2000	1.40	-0.1500	0.88	**0.440**
**RPG**	-1.1000	1.20	-0.9100	0.36	0.000
**Time Wasters**	0.0510	1.00	0.0490	0.96	0.000
**Action Multiplayer**	1.0000	1.20	0.8500	0.4	0.000
**Shopping**	1.1000	1.10	1.0000	0.31	**1.200**
**Auction Websites**	1.5000	1.20	1.2000	0.22	**0.570**
**Gambling**	1.9000	1.10	1.7000	0.09	**0.053**
**Social networking**	2.7000	1.00	2.6000	< 0.01	**0.590**
**Sports**	-2.1000	1.10	-1.8000	0.07	0.000
**Pornography**	2.1000	1.10	1.9000	0.05	**1.200**
**Messaging**	0.2000	0.91	0.2200	0.82	0.000
**Streaming media**	-0.6300	1.10	-0.6000	0.55	0.000
**Age x General Surfing**	-2.1000	0.79	-2.7000	< 0.01	0.000
**Age x Internet Gaming**	-0.4400	0.82	-0.5400	0.59	0.000
**Age x RPG**	1.4000	0.75	1.9000	0.06	**0.310**
**Age x Time Wasters**	1.1000	0.69	1.6000	0.1	0.000
**Age x Action Multiplayer**	-0.3000	0.77	-0.4000	0.69	0.000
**Age x Shopping**	-1.2000	0.81	-1.4000	0.15	0.000

**Age x Gambling**	-0.2000	0.87	-0.2200	0.82	0.000
**Age x Auction Websites**	1.1000	0.86	1.3000	0.19	0.000
**Age x Social Networking**	0.0015	0.76	0.0019	1	0.000
**Age x Sports**	1.7000	0.75	2.2000	0.03	0.000
**Age x Pornography**	-0.8500	0.69	-1.2000	0.21	0.000
**Age x Messaging**	0.7200	0.66	1.1000	0.28	0.000
**Age x Streaming media**	0.5800	0.76	0.7600	0.45	**0.310**
**Gender x General Surfing**	-0.6800	0.85	-0.8100	0.42	0.000
**Gender x Internet Gaming**	1.4000	0.99	1.4000	0.17	0.000
**Gender x RPG**	0.2600	0.92	0.2900	0.78	0.000
**Gender x Time Wasters**	-1.0000	0.79	-1.3000	0.2	0.000
**Gender x Action Multiplayer**	-0.8300	1.00	-0.8100	0.42	0.000
**Gender x Shopping**	1.2000	0.88	1.4000	0.18	0.000
**Gender x Gambling**	-1.0000	0.86	-1.2000	0.22	0.000
**Gender x Auction Websites**	-1.8000	0.86	-2.1000	0.04	0.000
**Gender x Social Networking**	-1.8000	0.84	-2.1000	0.03	0.000
**Gender x Sports**	0.6800	0.94	0.7300	0.47	0.000
**Gender x Pornography**	0.3300	1.10	0.3100	0.75	0.000
**Gender x Messaging**	-0.7300	0.74	-0.9800	0.33	0.000
**Gender x Streaming media**	0.7100	0.82	0.8700	0.39	0.000

T value - coefficient of linear regression; Pr(>|t|) - p-value of linear regression; Lasso - least absolute shrinkage and selection operator; RPG - Role Playing games; PADUA: Padua Inventory-Revised Checking; BIS - Barratt Impulsiveness Scale 11; ADHD - Attention Deficit Hyperactivity Disorder; GAD – Generalized Anxiety disorder; OCD – Obsessive-Compulsive disorder;

Figure S1 – Exploratory plots of Internet addiction vs internet activity coloured by gender

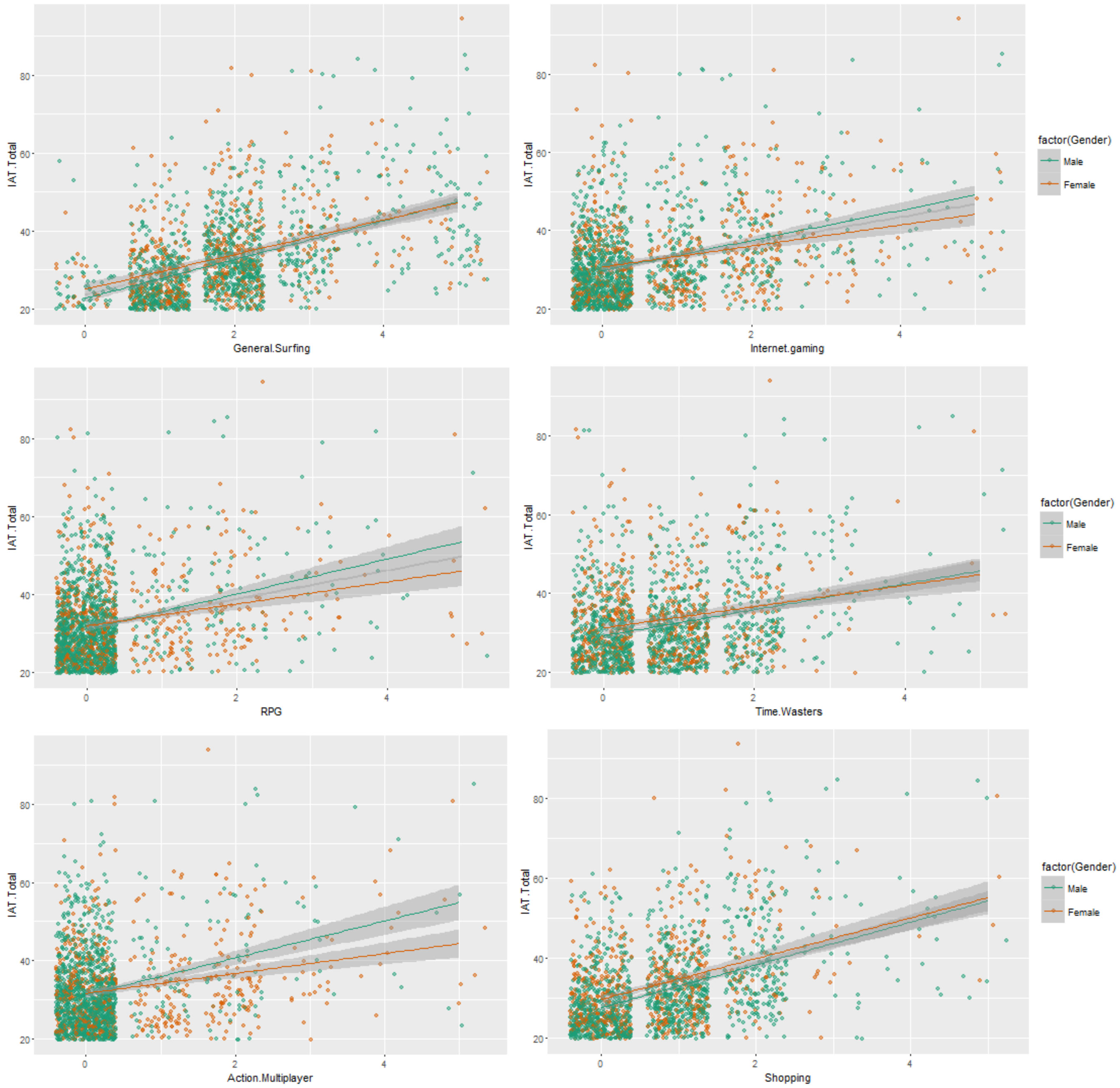

Figure S1 – (continuation) Exploratory plots of Internet addiction vs internet activity coloured by gender

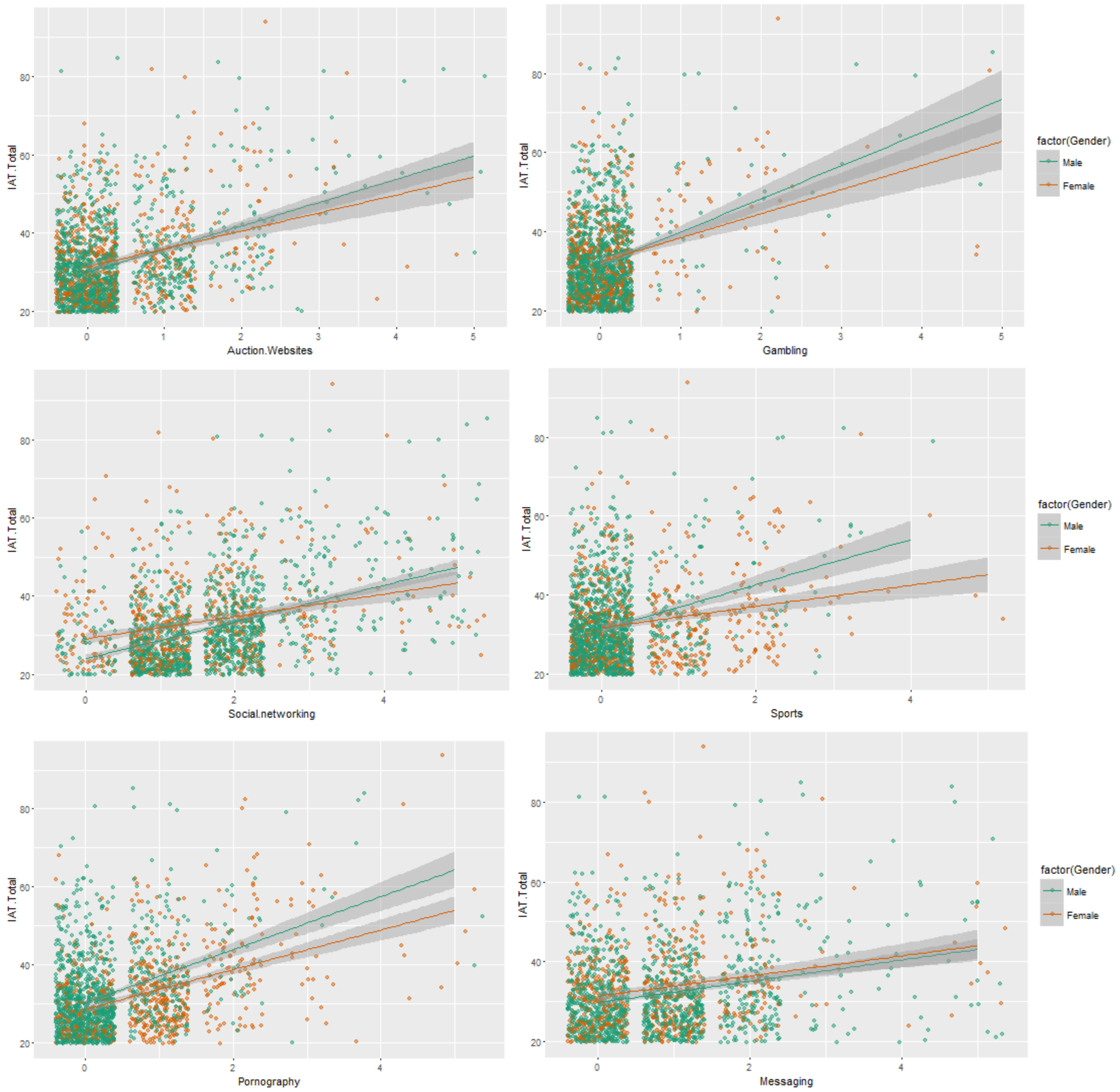

Figure S2 – Exploratory plots of Internet addiction vs internet activity coloured by age

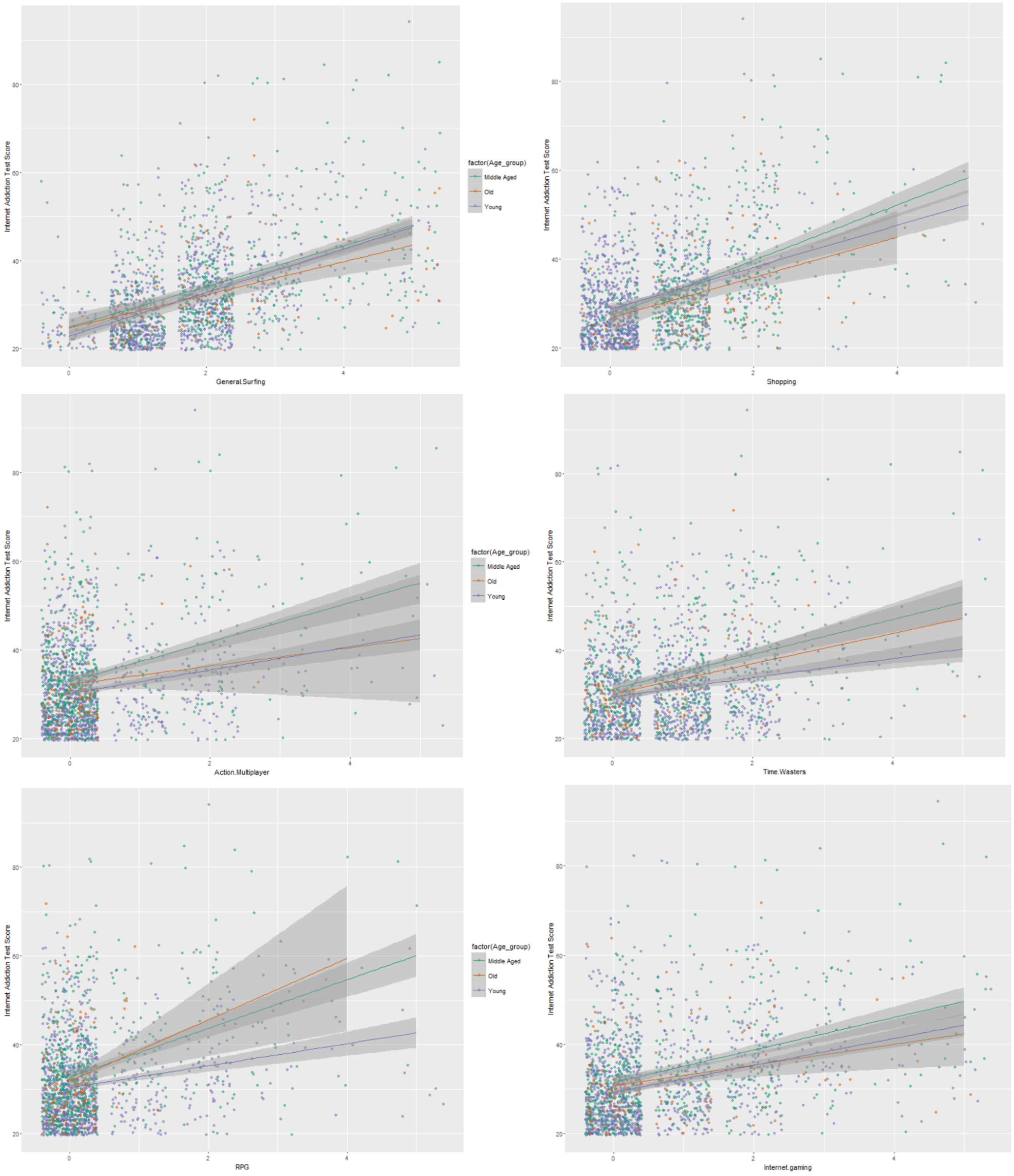

Figure S2 – (continuation) Exploratory plots of Internet addiction vs internet activity coloured by age

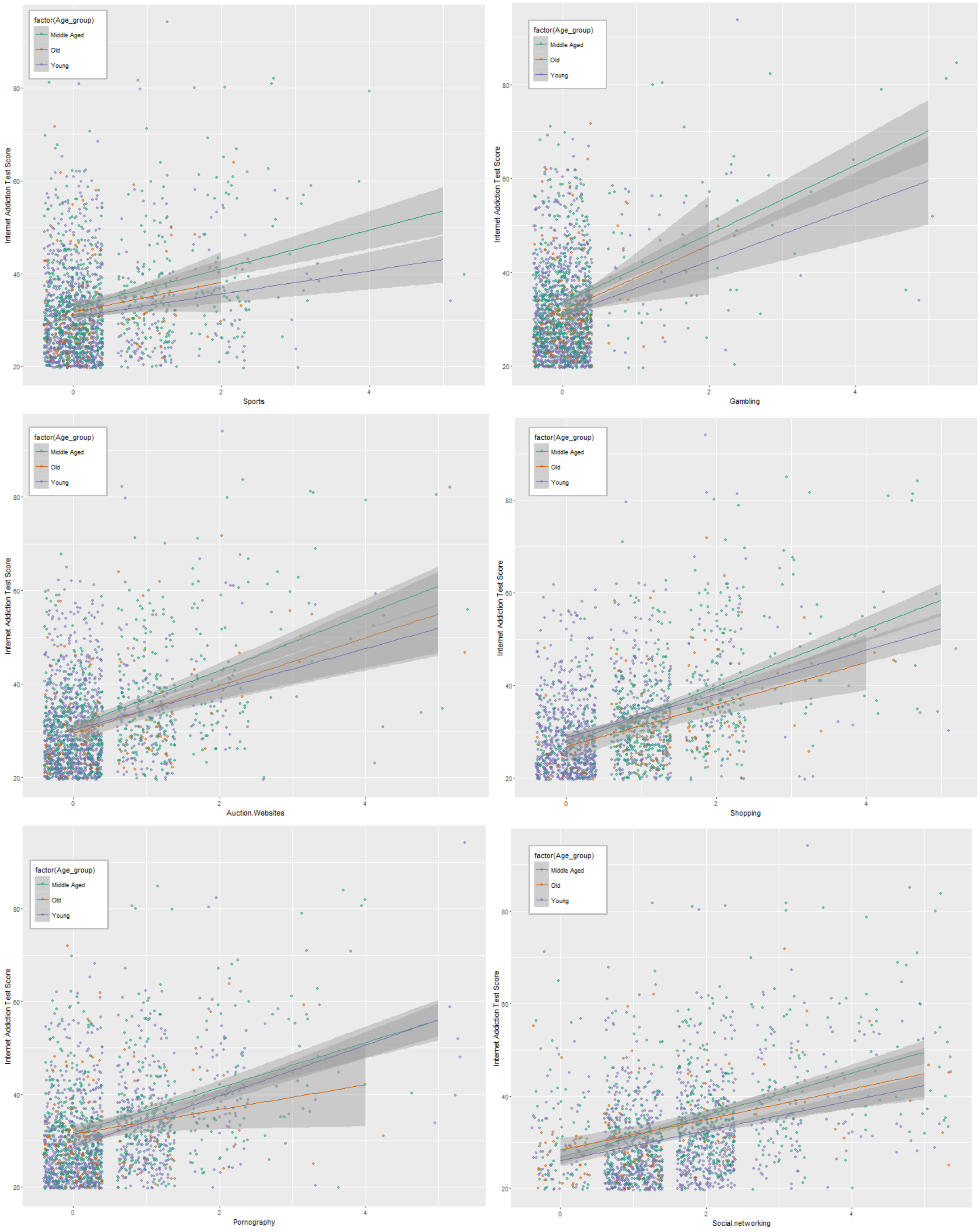

Figure S3 – Exploratory plot of Internet addiction vs diagnosis coloured by age group

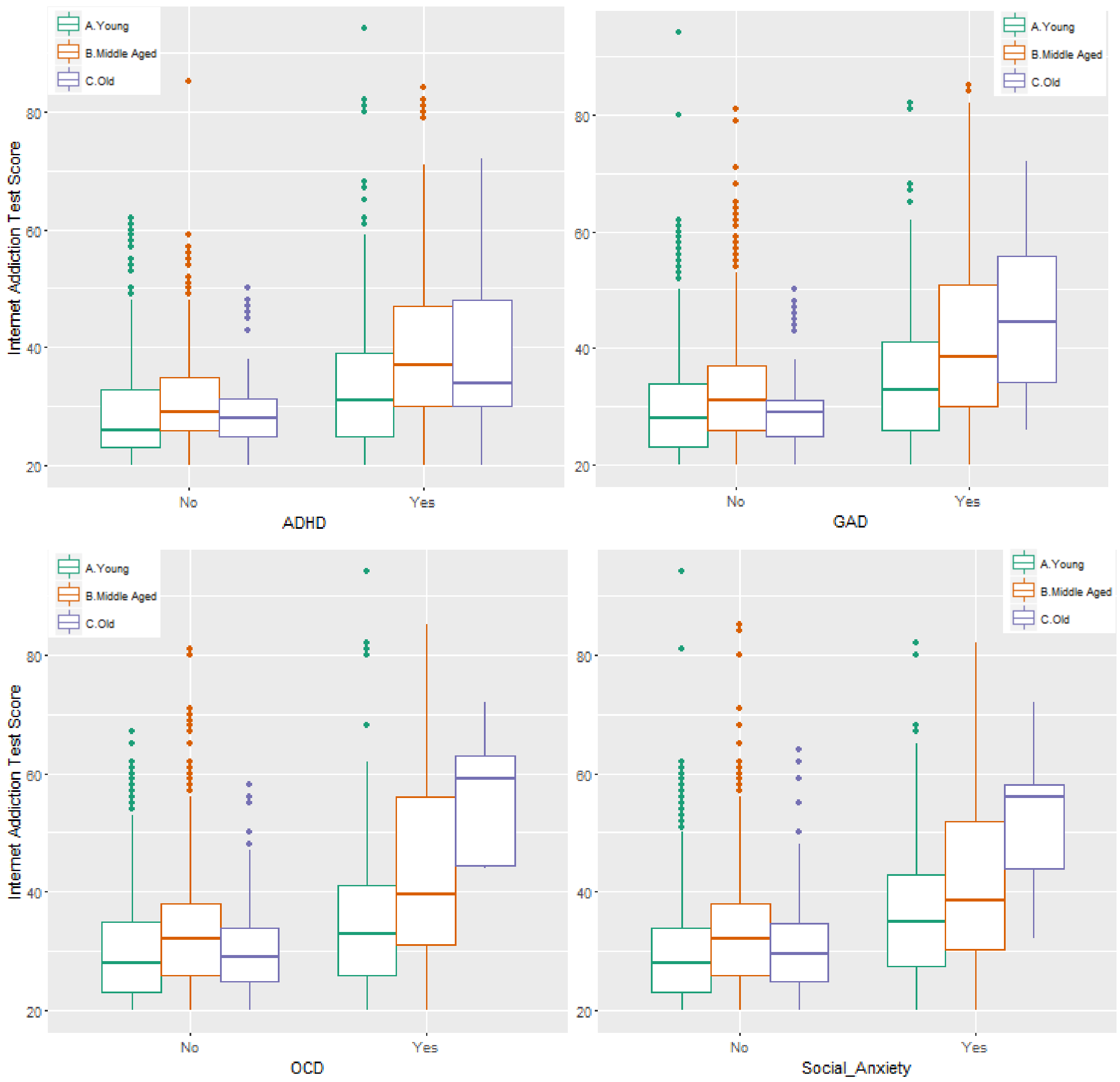

ADHD - Attention Deficit Hyperactivity Disorder; GAD – Generalized Anxiety disorder; OCD – Obsessive-Compulsive disorder;

R script

```
#Preparing data set
df <- read.csv("~/Psychiatry Research/ICOCS/df.csv", head = TRUE, stringsAsFactors = FALSE)
df <- data.table(df)
df$Site <- as.factor(df$Site)
df <- df %>% dplyr::select(Site:Education, IAT.Total, BISAI, BISMI, BISNI, PADUA.COWC:ADHD.Y.N)
df <- df %>% mutate(ASRS = ASRS1 + ASRS2 +ASRS3 + ASRS4 +ASRS5 + ASRS6) %>% dplyr::select(-ASRS1:-ASRS18)
df <- df %>% dplyr::select(Site:Education, -Race,  BISAI:PADUA, ASRS, GAD.Y.N:ADHD.Y.N,-APD.Y.N, -OCPD.Y.N,
                        General.Surfing:NEIU, IAT.Total, ASRS, ADHD.Y.N , General.Surfing:Streaming.media)

#-General.Surfing:-NEIU
df$ADHD.Y.N <- as.factor(df$ADHD.Y.N); df$GAD.Y.N <- as.factor(df$GAD.Y.N); df$Soc.Anx.Y.N <-
as.factor(df$Soc.Anx.Y.N)
df$OCD.Y.N <- as.factor(df$OCD.Y.N); ##df3$OCPD.Y.N <- as.factor(df3$OCPD.Y.N); df3$APD.Y.N <-
as.factor(df3$APD.Y.N)
df$Gender <- as.factor(df$Gender)  ; df$Race.Binary <- as.factor(df$Race.Binary); df$Education <-
as.factor(df$Education)
df$Relationship <- as.factor(df$Relationship) ; df$Sex.Orient <- as.factor(df$Sex.Orient)

df <- df %>% mutate(BIS = BISAI + BISMI + BISNI)
df <- df %>% dplyr::select(-BISAI:-PADUA.IHSO, -ASRS, -NEIU, -EIU)
df <- df %>% filter(Age>17)
df <- df %>% filter(Gender != 2)
df <- df %>% filter(complete.cases(.))
#df <- within(df, (IAT50= ifelse(IAT.Total < 50, 0, 1)))
df$Sex.Orient <- as.numeric(df$Sex.Orient); df <- within(df, (Sex.Orient= ifelse(Sex.Orient < 2, 1, 2)));df$Sex.Orient <-
as.factor(df$Sex.Orient)
df4 <- df
df4$Gender <- as.numeric(df4$Gender)

df5 <- df4 %>% mutate(AgexGS = Age * General.Surfing) %>%
          mutate(AgexIG = Age * Internet.gaming) %>%
           mutate(AgexRPG = Age * RPG) %>%
           mutate(AgexTW = Age * Time.Wasters) %>%
           mutate(AgexAM = Age * Action.Multiplayer) %>%
           mutate(AgexSh = Age * Shopping) %>%
           mutate(AgexG = Age * Gambling) %>%
          mutate(AgexAW = Age * Auction.Websites) %>%
          mutate(AgexSN = Age * Social.networking) %>%
          mutate(AgexSp = Age * Sports) %>%
          mutate(AgexPorn = Age * Pornography) %>%
          mutate(AgexMess = Age * Messaging) %>%
          mutate(AgexSM = Age * Streaming.media) %>%
          mutate(GenderxGS = Gender * General.Surfing) %>%
          mutate(GenderxIG = Gender * Internet.gaming) %>%
           mutate(GenderxRPG = Gender * RPG) %>%
           mutate(GenderxTW = Gender * Time.Wasters) %>%
           mutate(GenderxAM = Gender * Action.Multiplayer) %>%
           mutate(GenderxSh = Gender * Shopping) %>%
           mutate(GenderxG = Gender * Gambling) %>%
          mutate(GenderxAW = Gender * Auction.Websites) %>%
          mutate(GenderxSN = Gender* Social.networking) %>%
```

```
          mutate(GenderxSp = Gender * Sports) %>%
          mutate(GenderxPorn = Gender * Pornography) %>%
          mutate(GenderxMess = Gender * Messaging) %>%
          mutate(GenderxSM = Gender * Streaming.media)

df6 <- dplyr::select(df5, Site, Gender:Race.Binary, Relationship, Sex.Orient,
Education,General.Surfing:Streaming.media,IAT.Total,AgexGS:GenderxSM,
                  BIS, PADUA, ADHD.Y.N, OCD.Y.N, Soc.Anx.Y.N, GAD.Y.N)
#Linear regression example
fit <- lm(IAT.Total ~ ., data = df6)
summary(fit)
#plot(fit)

#Cross-validating RMSE as function of variable number
#First creating a predict.rgsubsets function
par(mfrow=c(2,2))

  predict.regsubsets = function(object, newdata, id, ...) {
    form = as.formula(object$call[[2]])
    mat = model.matrix(form, newdata)
    coefi = coef(object, id = id)
    mat[, names(coefi)] %*% coefi
  }

set.seed(23)
folds=sample(rep(1:10,length=nrow(df6)))
cv.errors=matrix(NA,10,56)
for(k in 1:10){
  cv.fit=regsubsets(IAT.Total ~ ., data=df6[folds!=k,],nvmax=56, method="backward")
  for(i in 1:56){
    pred=predict(cv.fit,df6[folds==k,],id=i)
    cv.errors[k,i]=mean( (df6$IAT.Total[folds==k]-pred)^2)
  }
}
rmse.cv=sqrt(apply(cv.errors,2,mean))

plot(rmse.cv,pch=19,type="b")


# This is the code for model comparisons
rmse.lm.mat <- matrix(0, 0, 1); colnames(rmse.lm.mat) <- c("RMSE.lm")
rmse.lasso.mat <- matrix(0, 0, 1); colnames(rmse.lasso.mat) <- c("RMSE.lasso")
rmse.enet.mat <- matrix(0, 0, 1); colnames(rmse.enet.mat) <- c("RMSE.enet")
rmse.rf.mat <- matrix(0, 0, 1); colnames(rmse.rf.mat) <- c("RMSE.rf")
rmse.ridge.mat <- matrix(0, 0, 1); colnames(rmse.ridge.mat) <- c("RMSE.ridge")
k = 50
set.seed(23)
for (i in 1:k) {
  inTrain <- createDataPartition(y = df6$IAT.Total,
                              p = 0.75, list = FALSE)
  training <- df6[as.vector(inTrain), ]
  testing <- df6[-as.vector(inTrain), ]
```

```
  training <- training %>% mutate_each_(funs(scale), vars =
c("Age","General.Surfing","Internet.gaming","RPG","Time.Wasters","Action.Multiplayer",

"Shopping","Auction.Websites","Gambling","Social.networking","Sports","Pornography",

"Messaging","Streaming.media","AgexIG","AgexRPG","AgexTW","AgexAM","AgexSh","AgexG",

"AgexAW","AgexSN","AgexSp","AgexPorn","AgexMess","AgexSM","GenderxIG","GenderxRPG",

"GenderxTW","GenderxAM","GenderxSh","GenderxG","GenderxAW","GenderxSN","GenderxSp",
                                        "GenderxPorn","GenderxMess","GenderxSM","PADUA","BIS"))

  testing <- testing %>% mutate_each_(funs(scale), vars =
c("Age","General.Surfing","Internet.gaming","RPG","Time.Wasters","Action.Multiplayer",
"Shopping","Auction.Websites","Gambling","Social.networking","Sports","Pornography",
"Messaging","Streaming.media","AgexIG","AgexRPG","AgexTW","AgexAM","AgexSh","AgexG",
"AgexAW","AgexSN","AgexSp","AgexPorn","AgexMess","AgexSM","GenderxIG","GenderxRPG",
"GenderxTW","GenderxAM","GenderxSh","GenderxG","GenderxAW","GenderxSN","GenderxSp",
"GenderxPorn","GenderxMess","GenderxSM","PADUA","BIS"))

  control <- trainControl(method="cv", number=10)
  modFit<- train(IAT.Total ~. , data = as.data.frame(training),
             metric = "RMSE",
             method = "lm",
            trControl = control)

  lm.test.prob = predict(modFit, newdata = testing);
  rmse.lm <- sqrt(mean((testing$IAT.Total - predict(modFit,testing))^2))
  rmse.lm.mat <- rbind(rmse.lm.mat, rmse.lm)
  control <- trainControl(method="cv", number=10)
  modFit<- train(IAT.Total ~. , data = as.data.frame(training),
             metric = "RMSE",
             method = "lasso", tuneLength = 10,
             trControl = control)
  lasso.test.prob = predict(modFit, newdata = testing);
  rmse.lasso <- sqrt(mean((testing$IAT.Total - predict(modFit,testing))^2))
  rmse.lasso.mat <- rbind(rmse.lasso.mat, rmse.lasso)
  control <- trainControl(method="cv", number=10)
  modFit<- train(IAT.Total ~. , data = as.data.frame(training),
             metric = "RMSE",
             method = "enet", tuneLength = 10,
             trControl = control)
  enet.test.prob = predict(modFit, newdata = testing);
  rmse.enet <- sqrt(mean((testing$IAT.Total - predict(modFit,testing))^2))
  rmse.enet.mat <- rbind(rmse.enet.mat, rmse.enet)

  control <- trainControl(method="cv", number=10)
  modFit<- train(IAT.Total ~. , data = as.data.frame(training),
             metric = "RMSE",
             method = "ridge", tuneLength = 10,
             trControl = control)

  ridge.test.prob = predict(modFit, newdata = testing);
```

```
  rmse.ridge <- sqrt(mean((testing$IAT.Total - predict(modFit,testing))^2))
  rmse.ridge.mat <- rbind(rmse.ridge.mat, rmse.ridge)

  control <- trainControl(method="cv", number=10)
  modFit<- train(IAT.Total ~. , data = as.data.frame(training),
               metric = "RMSE",
               method = "rf", tuneLength = 10,
               trControl = control)

  rf.test.prob = predict(modFit, newdata = testing);
  rmse.rf <- sqrt(mean((testing$IAT.Total - predict(modFit,testing))^2))
  rmse.rf.mat <- rbind(rmse.rf.mat, rmse.rf)



  results <- cbind(rmse.lm.mat, rmse.lasso.mat, rmse.enet.mat, rmse.ridge.mat, rmse.rf.mat)

  cat(i," ")
}


#Now we fit a lasso model; for this we use the default `alpha=1`
x=model.matrix(IAT.Total ~ ., data=df6)
y=df6$IAT.Total

fit.lasso=glmnet(x,y)

cv.lasso=cv.glmnet(x,y)
plot(cv.lasso)
plot(fit.lasso,xvar="lambda",label=TRUE)
plot(fit.lasso,xvar="dev",label=TRUE)
coef(cv.lasso)

regr_tab <- data.frame(summary(fit)$coefficients) # grab the coefficients names
colnames(regr_tab) <- colnames(summary(fit)$coefficients) # get the p-vals and delete/round up digits

regr_tab[ ,4] <- ifelse(regr_tab[ ,4] < .001, "< 0.001",
                     ifelse(regr_tab[ ,4] < .01, "< 0.01",
                         round(regr_tab[ ,4], 2)))

regr_tab[ ,1] <- signif(regr_tab[ ,1], digits = 2)
regr_tab[ ,2] <- signif(regr_tab[ ,2], digits = 2)
regr_tab[ ,3] <- signif(regr_tab[ ,3], digits = 2)

LASSO.coefficient <- as.vector(coef(cv.lasso))
LASSO.coefficient <- as.data.frame(LASSO.coefficient);LASSO.coefficient <- LASSO.coefficient[-2,];LASSO.coefficient
<- as.data.frame(LASSO.coefficient)
regr_tab <- cbind(regr_tab, LASSO.coefficient)
regr_tab[ ,5] <- signif(regr_tab[ ,5], digits = 2)
regr_tab
```
